# Improving methods to evaluate the impacts of plant invasions: lessons from 40 years of research

**DOI:** 10.1093/aobpla/plv028

**Published:** 2015-03-30

**Authors:** Kerry Bohl Stricker, Donald Hagan, S. Luke Flory

**Affiliations:** 1 Agronomy Department, University of Florida, Gainesville, FL 32611, USA; 2 School of Agricultural, Forest, and Environmental Sciences, Clemson University, Clemson, SC 29634, USA

**Keywords:** Biological invasions, experimental methods, invasive plant, non-native species, observational methods, predictive modelling

## Abstract

Methods used to evaluate the ecological impacts of biological invasions vary widely from broad-scale observational studies to removal experiments in invaded communities and experimental additions in common gardens and greenhouses. Different methods provide information at diverse spatial and temporal scales with varying levels of reliability. Thus, here we provide a synthetic and critical review of the methods used to evaluate the impacts of plant invasions and provide recommendations for future research. We review the types of methods available and report patterns in methods used, including the duration and spatial scale of studies and plant functional groups examined, from 410 peer-reviewed papers published between 1971 and 2011. We found that there has been a marked increase in papers published on plant invasion impacts since 2003 and that more than half of all studies employed observational methods while <5 % included predictive modelling. Most of the studies were temporally and spatially restricted with 51 % of studies lasting <1 year and almost half of all studies conducted in plots or mesocosms <1 m^2^. There was also a bias in life form studied: more than 60 % of all studies evaluated impacts of invasive forbs and graminoids while <16 % focused on invasive trees. To more effectively quantify invasion impacts, we argue that longer-term experimental research and more studies that use predictive modelling and evaluate impacts of invasions on ecosystem processes and fauna are needed. Combining broad-scale observational studies with experiments and predictive modelling may provide the most insight into invasion impacts for policy makers and land managers seeking to reduce the effects of plant invasions.

## Abstract (precis)

Here the authors review the research methods used to measure the ecological effects of non-native plant invasions. In their synthesis they find that although the number of studies on invasion impacts has increased markedly in recent years, there is a lack of experimental studies, a bias among invader functional groups, and relatively few studies on ecosystem effects of invasions. They recommend utilization of longer-term studies that combine broad-scale observations, experimental manipulations, and predictive modelling across diverse invader functional groups and affected ecosystems to provide more comprehensive insight into the impacts of plant invasions.

## Introduction

Globalization has resulted in dramatic increases in intentional and accidental introductions of plants to non-native ranges. Such introductions are often innocuous but at times result in widespread, ecologically damaging invasions (Simberloff *et al.* 2013). Recent syntheses of the growing body of work on the ecological impacts of non-native plant invasions indicate that they can lead to reductions in plant community diversity and performance, inhibition of succession in forests and other ecosystems and alteration of nutrient cycling, hydrology and fire regimes, among other effects (Mack *et al*. 2000; Ehrenfeld 2003, 2010; Vilà *et al*. 2011; Pyšek *et al*. 2012). However, given the rapid rate of non-native plant introductions (Hulme *et al*. 2009) and our current limitations in making generalizations regarding their impacts (Hulme *et al.* 2013), additional research to quantify the effects of invasions is needed. Such studies will help motivate protection and restoration of natural areas and inform prioritization of species for management (Parker *et al*. 1999; Simberloff *et al*. 2013).

Although there has been considerable interest in synthesizing the growing body of work on the ecological effects of plant invasions (Pyšek *et al*. 2012), few studies have critically evaluated patterns in research methodology (e.g. Parker *et al*. 1999; Hulme *et al*. 2013). Research methods used to evaluate impacts of plant invasions can vary widely, with different approaches providing information at diverse spatial and temporal scales, thereby influencing data reliability and resulting inferences (Kumschick *et al*. 2015). An early review of approaches used to quantify the impacts of all invasive taxa found that of the relatively small number of studies undertaken, most were purely correlative and only 8 % combined both observational and experimental components (Parker *et al.* 1999). Such combination studies can be particularly informative because they provide the most realistic measures of invasion impacts in natural settings while also elucidating cause and effect. At the time of their review, Parker *et al.* (1999) identified a lack of theoretical and mathematical models in impact studies and documented few studies addressing ecosystem-level consequences of plant invasions. They called for more studies that synthesize impacts of invasive taxa and additional work to evaluate impacts at a variety of spatial and temporal scales, arguing that such efforts would more effectively characterize, predict and generalize invasive species impacts. Since Parker *et al.*'s (1999) evaluation of invasion impacts, there has been an impressive increase in impact studies published, yet the field of invasion biology continues to be criticized for its inability to generate clear conclusions regarding the true effects of invasions (Hulme *et al.* 2013). Therefore, our objectives here were to review the wide variety of methods used to evaluate impacts of terrestrial plant invasions and identify patterns in how impacts have been assessed, thereby helping to guide future invasive plant impact research. Although Hulme *et al.* (2013) previously examined study biases in terms of life form, geography and focal species in field studies, our study is the first to evaluate the full range of approaches used to study the ecological consequences of plant invasions.

Here we provide a brief review of the methods used to evaluate plant invasion impacts and then report patterns in how impacts have been assessed from 410 peer-reviewed papers published on terrestrial plant invasion impacts between 1971 and 2011. Our first aim was to determine whether there has been an increase in the use of predictive modelling and experimental methods over time and whether observational and experimental methods are increasingly being used in combination. Second, we evaluated the spatial scale and temporal duration of studies and how study duration varied among research methods. Third, we looked for how research effort was allocated across invasive plant functional groups and among the types of effects measured over time. Fourth, we sought to determine how patterns in invasion impact research have changed since the review by Parker *et al.* (1999). Finally, we provide recommendations for improving future research. We ultimately seek to identify trends in plant invasion impact research methodology and to highlight the advantages of coupling observational studies with experimental and/or modelling studies to provide more reliable data for prioritizing management and informing policy-making decisions.

## Methods Used to Evaluate Impacts of Plant Invasions

Studies evaluating the impacts of plant invasions can be observational, experimental, modelling-based or some combination of techniques. Observational studies often document differences among invaded and adjacent uninvaded areas (e.g. Standish *et al*. 2004) or less commonly, before and after an invasion has occurred (e.g. Kwiatkowska *et al*. 1997). Because such studies generally require lower input of resources relative to experimental research, they often can be conducted at a larger scale and thus present an increased potential for generalization. Observational studies can provide a broad survey of differences among communities and ecosystems based on invaded or uninvaded status, the abundance or density of the invader or the time since invasion. However, it is often not possible to disentangle cause and effect (MacDougall and Turkington 2005; Bauer 2012). That is, observed differences may be due to the impact of the invasion itself, or alternatively, some prior disturbance or change in the system may have altered biodiversity or ecosystem processes and simultaneously promoted the invasion (MacDougall and Turkington 2005). In such cases, the invasion may be a secondary symptom of an underlying change, not a direct cause of the community or ecosystem impact. Moreover, in systems invaded by more than one non-native plant species, it is usually not possible to determine the relative contribution of each species to observed effects. Despite their limitations, the primary benefit of observational studies is that they document patterns across broad-scale realistic natural conditions that can then be used to inform further experimental and modelling studies.

**Figure 1.**
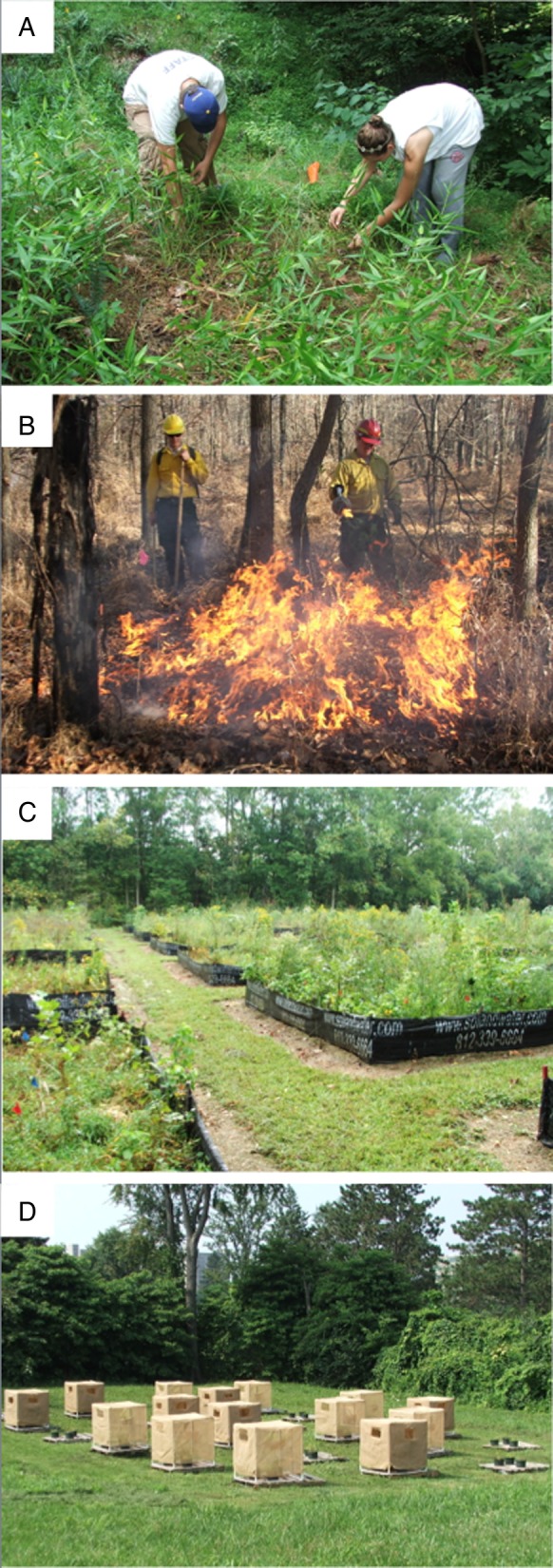
Examples of experimental methods to evaluate invasion impacts: (A) removal by hand, (B) treatment with prescribed fire, (C) addition of an invasive plant in a common garden and (D) addition of an invasive species in outdoor mesocosms under multiple shade treatments. All photos S.L. Flory.

Studies that use experimental approaches to evaluate the impacts of plant invasions include experimental removal (Fig. 1A and B) or addition (Fig. 1C and D) of the target species. Experimental removal studies indirectly indicate plant invasion impacts by evaluating how the community responds once the invasive species has been removed (e.g. Alvarez and Cushman 2002; Gratton and Denno 2005; Flory and Clay 2009; Spellman and Wurtz 2011). Removal of invasive plants can be accomplished with mechanical (pulling, mowing, string trimming, Fig. 1A) or chemical (pre- or post-emergent herbicide) treatments, biological control agents or by prescribed burning (Fig. 1B). The advantage of removal studies is that by experimentally removing the invasive plant it is relatively straightforward to interpret differences among invaded and experimentally treated plots. In addition, there are fewer ethical considerations than with experimental addition studies. However, although it may be possible to remove the invasive plant itself, there may be lasting (i.e. legacy) effects of the invasion on soil chemistry or microbial communities (Marchante *et al*. 2009), the response of the native community may be delayed, native species may respond to the disturbance caused by the removal of invasive plants, or other invasive plant species might colonize the site (Mack and Lonsdale 2002; Ogden and Rejmánek 2005; Mau-Crimmins 2007). Furthermore, the method used to remove the invasive plant may influence the native community response. For example, application of a grass-specific herbicide effectively removed an invasive grass and allowed native forbs and trees to return, whereas hand-weeding inhibited tree and fern recovery (Flory and Clay 2009). Given the potential difficulty in interpreting responses to experimental removal treatments, it was recently recommended to simultaneously establish plots where the invader is removed and at the same time to remove natives from uninvaded plots (Kumschick *et al*. 2015). Coupled with observations of invaded and uninvaded areas, such a design would allow for evaluation of possible disturbance effects associated with the removal treatments and inform the success of restoration efforts.

Experimental addition studies can be conducted in the field, greenhouse, common garden or lab, and might include the addition of seed, seedlings or plant litter or other parts of the non-native invasive plant (e.g. Vilà *et al*. 2004; Maron and Marler 2008; Flory and Clay 2010). Addition studies are advantageous in that they often include experimental controls such that all differences in invaded plots or mesocosms can be attributed to the invader (Fig. 1C). They can also be conducted under particular environmental conditions (Fig. 1D) or disturbance regimes, and in native communities with planned species compositions (Maron and Marler 2008). However, there are ethical concerns with introducing an invasive plant, including the risk of escape to surrounding natural areas. Therefore, studies are often confined to only one or a few sites near where the invasion already occurs naturally or studies may be restricted to greenhouse, growth chamber, lab or outdoor mesocosm settings. Additionally, the timing and duration of studies may be limited due to concerns about dispersal into surrounding natural areas, experiments might only include certain plant life history stages such as non-reproductive juveniles that have low risk of escape, and invaders may be added at unrealistically low densities and therefore may underestimate invasion impacts. Such factors can limit the realism of experimental addition studies.

Studies that focus on or incorporate modelling often attempt to predict long-term changes based on data fitted from shorter term observational or experimental studies (Parker *et al*. 1999; Gallien *et al*. 2010). Some modelling studies involve the use of Geographic Information Systems (GIS) to extrapolate the results from short-term, small-scale studies to assess potential impact at the landscape scale (Peterson *et al*. 2003; Lindsay *et al*. 2011). Other studies are purely quantitative, being based on empirically derived equations that model some aspects of invasion (e.g. population dynamics; interspecific interactions, alterations to nutrient cycling processes; Harrison *et al*. 2006; Gómez-Aparicio and Canham 2008; Atwood *et al*. 2010; Takahashi *et al*. 2011). Modelling methods allow researchers to address research questions that would otherwise be difficult—if not impossible—due to logistical, economic or ethical constraints (Jackson *et al*. 2000). Additionally, their predictive ability makes them attractive as decision-making tools (Schmolke *et al*. 2010). However, the utility of modelling studies is often limited by the quality or completeness of available data (Peterson *et al*. 2003; Radosevich *et al*. 2003) and the reliance on oversimplified assumptions about complex ecological processes (Gallien *et al*. 2010).

## Database Compilation and Statistical Analysis

To compile our database of plant invasion impact studies, we used a combination of online search tools and primary literature. First, we conducted comprehensive searches of ISI Web of Knowledge (http://wokinfo.com) and Google Scholar (http://scholar.google.com) in March and April 2012 to identify potential primary literature on plant invasion impacts. We used all possible combinations of search terms associated with plant invasion impacts such as invas* plant* impact* and non-native plant* effect*, among many others. In addition, we searched the references in review papers on invasion effects including Parker *et al.* (1999), Ehrenfeld (2010), Powell *et al.* (2011), Simberloff (2011) and Vilà *et al.* (2011). Papers published from 1971 through 2011 were collected that specifically quantified the impacts or effects of plant invasions on biological communities or ecosystem properties or processes. All possible research methods, from field observations and removal studies to experimental introductions in the lab and greenhouse, and all study locations and plant types were included in our database. We considered all temporal and spatial scales, but excluded studies that focused only on economic impacts, mechanisms of invasions or management. Our search was restricted to terrestrial non-native plants that were considered invasive (i.e. ecologically problematic).

We were specifically interested in the proportion of research effort that was focused on different methods and techniques, and papers often included multiple separate studies or experiments, so we evaluated and quantified all ‘studies’ within papers separately. Each study was classified as having used observational, experimental removal, experimental addition or modelling methods. The total number of papers and studies published per year using each method was tabulated and we calculated the percentage of papers that included both observational and experimental removal or addition studies. We also determined the duration of each study (years), functional group of the invasive plant (graminoid, forb, shrub, tree), spatial scale (<1 m^2^, 1 m^2^ to 4 m^2^, >4 m^2^), approach [field, greenhouse, lab (e.g. growth chamber), common garden or modelling] and impacted group evaluated (plants, invertebrates, ecosystem effect, vertebrates, microbes). For approaches, common garden studies included outdoor plots or experimental mesocosms. For impacted groups, ‘ecosystem’ impacts included alterations to nutrient pools or fluxes, fire regimes, decomposition or hydrology.

Patterns in the methods used to evaluate invasion impacts were analysed statistically using R (R Core Team 2013). The number of papers and studies published over time were analysed using general linear models. Data were natural log-transformed prior to analysis to increase conformity to normality and homoscedasticity assumptions when appropriate, and post hoc comparisons were performed using Tukey's honestly significant difference tests to adjust for multiple comparisons. Because the number of years represents count data, which are best analysed using generalized linear models, differences in the duration of studies by approach or functional group were analysed using Poisson regression. Finally, trends in study approaches used to assess invasive plant impacts on different impacted groups were analysed using contingency table analysis with Pearson's *χ*^2^ tests with simulated *P*-values based on 2000 replicates to account for cell counts fewer than five (Gotelli and Ellison 2004). Post hoc pairwise comparisons were achieved after the contingency table analysis using a Bonferroni correction to adjust *α* for multiple comparisons.

## Trends in Research Methods to Evaluate Invasion Impacts

**Figure 2.**
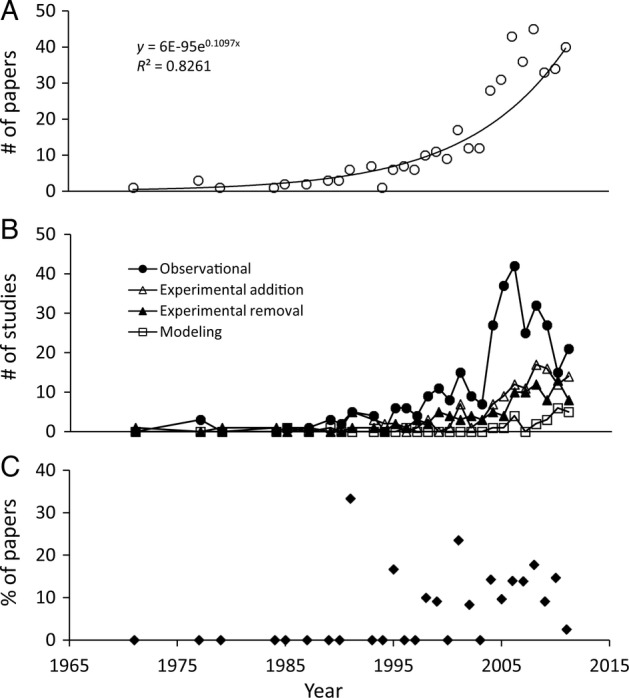
Number of papers published per year 1971–2011 that evaluated the ecological impacts of non-native plant invasions (solid line represents regression line) (A), the number of studies (some papers included multiple studies) that used each of four research methods (B) and the percentage of papers that included both an observational and an experimental study (C).

Our database contained a total of 410 papers and 576 studies published during 1971–2011 on the ecological effects of terrestrial plant invasions **[see Supporting Information]**. There was an exponential increase in the number of plant invasion impact papers published per year (*P* < 0.001, Fig. 2A), with a marked increase after 2003. The average number of papers published per year during 2003–11 was nearly four times greater than the yearly average during the previous 9 years. There was a significant interaction between study approach (observational, experimental addition, experimental removal or modelling) and time (*F*_3,104_ = 6.63, *P* < 0.001, Fig. 2B), indicating that the rate of increase in the number of studies varied across approaches. For example, the number of observational studies increased at a much greater rate than other methods since the late 1990s. There was also a dramatic increase in the number of studies using observational methods starting in 2003, a peak of more than 40 observational studies published in 2006, but then a steady decline up to 2011. In contrast, experimental removal, experimental addition and modelling studies have more steadily increased in number, particularly after 2000. Overall, the majority (55.6 %) of studies were observational, while 17.7 % of studies used experimental removal methods, 22.4 % were experimental addition and only 4.3 % utilized modelling. Papers that included both observational and either experimental removal or addition studies first appeared in 1992 (one out of three papers). On average, nearly 10 % of papers included both observational and experimental methods between 1995 and 2011, for a total of 6.3 papers per year on average, but there has been no increase in the percentage of such combination studies over time (*P* = 0.910, Fig. 2C).

**Figure 3.**
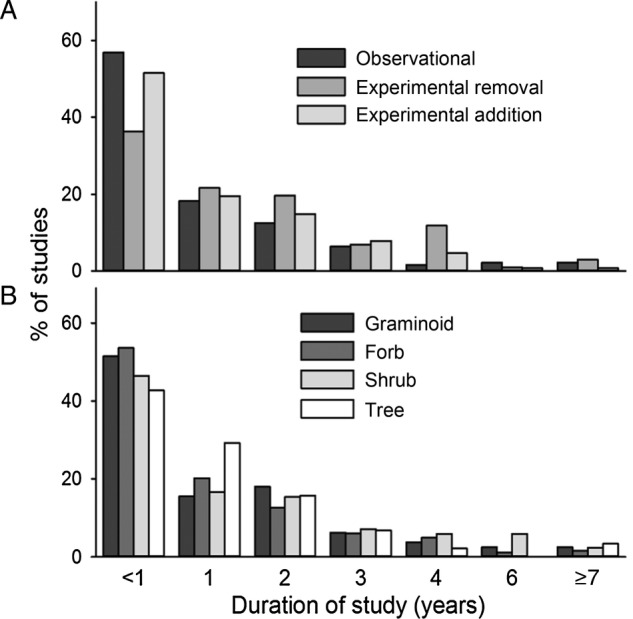
Percentage of observational, experimental removal and experimental addition studies (A) and graminoid, forb, shrub and tree studies (B) for each category of duration from <1 year to ≥7 years.

There were significant differences in the duration of studies based on approach (observational, experimental addition or experimental removal) ($\chi _2^2 = 26.3,$ *P* < 0.001). Experimental removal studies generally occur over a longer time interval than studies using other methods (*P* < 0.001, Fig. 3A) **[see** **Supporting Information****]** with more than 10 % lasting 4 years or more. More than 51 % of all studies on the impacts of plant invasions occurred over 1 year or less, including 57 % of observational studies, 36 % of experimental removal studies and 52 % of experimental addition studies. Only 7.9 % of studies lasted 4 or more years. There were also significant differences in the duration of studies among invasive plant functional groups ($\chi _3^2 = 9.06,$ *P* = 0.028), but only when studies examining invasive forbs versus those focused on invasive shrubs were compared, with studies on invasive shrubs more likely to be conducted over multiple years (Fig. 3B) **[see Supporting Information]**. Similar to the limitations on the duration of studies, many studies were limited in their spatial extent. Nearly half of all studies (49.3 %) were conducted at a scale of <1 m^2^ while 14.1 % used 1–4 m^2^ plots and less than one-third (31.1 %) of all studies used plots more than 4 m^2^. More than 80 % of all studies have been conducted in the field, while 9.5 % were conducted in the greenhouse, 4.7 % in the lab and 2.8 % utilized common garden designs.

**Figure 4.**
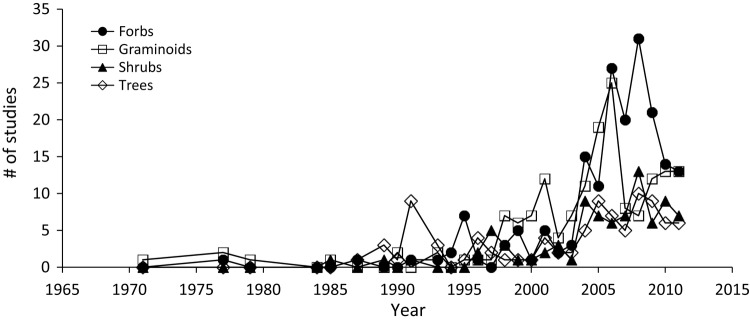
Number of studies conducted on invasive graminoids, forbs, shrubs and trees during 1971–2011.

From 1990 to 2011, there was a significant exponential increase in the number of studies conducted on the impacts of invasive plants across all functional groups (*F*_1,76_ = 120.5, *P* < 0.001; Fig. 4). There was little difference among functional groups until 1990, as few studies were conducted on plant invasion impacts prior to that year, but in the following years there were significant differences among functional groups (*F*_3,76_ = 3.48, *P* = 0.020), with more studies conducted on invasive graminoids and forbs than on shrubs (*P* = 0.03, *P* = 0.05, respectively). None of the other differences among functional groups were significant. From 2000 to 2011, when the vast majority (84 %) of all invasion impact studies were published, more than 52 % of studies were conducted on herbaceous species. During the same time period, there were on average 5.5 studies per year on invasive trees but more than 11.5 studies per year on forbs and 13.6 per year on invasive graminoids.

**Figure 5.**
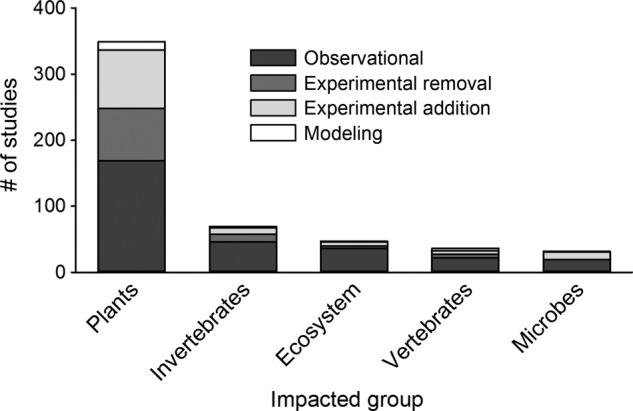
Number of observational, experimental removal, experimental addition and modelling studies that evaluated the impacts of invasions on different groups of organisms or ecosystem processes.

We also found significant differences in study approaches used to assess the impacts of invasive plants on different impacted groups (*χ*^2^ = 33.1, *P* = 0.002; Fig. 5). The majority (60.6 %) of plant invasion impact studies have focused on their effects on other plants, 12 % on invertebrates, 8.2 % on ecosystem effects, 6.3 % on vertebrates and only 5.5 % on microbes. The number of studies that evaluated the impacts of invasive plants on other plants was significantly greater than the number of studies evaluating plant invasion impacts on ecosystem processes (*χ*^2^ = 13.3, *P* = 0.006; Bonferroni-corrected *α* = 0.017). Of the studies that evaluated the impacts of invasions on other plants, nearly half of the studies used observational methods while 23 % used experimental removal and 25 % experimental addition. In contrast, more than 77 % of the studies on ecosystem effects used observational methods and few used experimental removal (8.5 %) or addition (12.8 %). Similarly, over 60 % of studies that quantified the effects of invasions on invertebrates, vertebrates and microbes used observational methods. A total of 77 studies have simultaneously evaluated multiple groups, most commonly plants and ecosystem effects (38 studies) and plants and invertebrates (11 studies).

## Synthesis and Recommendations

The steady increase in non-native plant introductions over time (Hulme *et al*. 2009) and the need to determine the consequences of invasions for native ecosystems has driven a significant increase in research on plant invasion impacts, particularly over the last decade (Pyšek and Richardson 2010). These efforts have greatly increased our understanding of how invasive species impact communities and ecosystems (Ehrenfeld 2010; Simberloff 2011; Pyšek *et al*. 2012), especially compared with when the topic was first comprehensively reviewed in the late 1990s (Parker *et al*. 1999). However, we found that patterns in the methodology and scale of research efforts have largely remained unchanged since the 1990s. The majority of studies continue to be limited in duration and spatial scale with much of the observed recent increase in impact studies attributable to purely observational studies. The relative proportions of experimental and combination studies conducted recently are also similar to those documented by Parker *et al.* in the late 1990s, despite the call for an increase in such studies. Furthermore, most of the studies have focused on the effects of herbaceous invasive plants and are often restricted to evaluation of impacts on other plants, while studies addressing ecosystem-level effects of invasions remain less well studied. The paucity of studies addressing ecosystem-level effects of invasive plants may hinder conclusions about impacts on ecosystem services, an area of great importance in terms of directing land management efforts.

The relatively high proportion of research effort focused on observational studies suggests that researchers are primarily concerned with broad patterns associated with invasions or that time and other resources required for experimental work continues to be a barrier. Observational approaches are quicker and simpler compared with experimental methods and are the logical first step in studying potential invasion impacts. However, although assumptions about invaders causing observed effects can be valid, at times the invasion may also be associated with community or ecosystem changes that are detrimental to native species and promote the invasive species. For example, disturbances such as fires or anthropogenic activities may cause declines in native plant species while allowing non-native invasive plants to colonize (Lake and Leishman 2004; Hill *et al*. 2005; MacDougall and Turkington 2005; Coffman *et al*. 2010). In such cases, accompanying experimental removal or addition studies would provide potentially critical information on the role of the target invasive species (Kumschick *et al*. 2015). For example, Alvarez and Cushman (2002) documented patterns of reduced native species diversity and abundance associated with English Ivy invasions and then showed that experimentally removing the ivy allowed native species to return. Similarly, Lee *et al.* (2012) found greater relative amounts of nitrate in soils across *Microstegium vimineum-*invaded forest stands compared with uninvaded areas and showed experimentally in the greenhouse and a common garden that the invader was responsible for increased nitrification potential.

Given the risk of introduction of new invaders or novel genetic material, experimental invasions should only be conducted in ecosystems that are already invaded by that particular species and propagules should be collected locally. State or federal laws prohibit the movement of some plant species and permits may be required to transport particular invasive species within or among states. Moreover, great care must be taken to prevent the escape of the invasive species from the experiment and spread to surrounding natural areas. Physical barriers such as erosion fencing or pre- or post-emergent herbicides may be effective for containing the experimentally added plant species. Careful monitoring of surrounding areas is necessary to ensure that the experiment is being effectively contained. Despite these logistical considerations, and because of their versatility and relative lack of complex interpretation, experimental addition studies provide perhaps the most effective method for evaluating how invasive plants are impacting ecosystem processes, particularly when coupled with observations of natural invasions and predictive modelling. Such ‘combination’ studies provide information on broad patterns in naturally invaded communities and also experimentally demonstrate that the invader is responsible for those changes (Kumschick *et al*. 2015). Studies using a combination of methods to evaluate plant invasion impacts have not significantly risen in recent years, indicating that further inclusion of both observational and experimental methods could greatly increase our understanding of both general patterns of changes in communities and ecosystems as a result of invasion and experimental evidence of cause and effect.

In Parker *et al.*'s (1999) review of the impacts of invasions, they provided three specific needs: additional research at multiple scales and levels of organization, more studies that synthesize available data and models to accompany empirical work. Over the last decade there has been a dramatic increase in the overall amount of invasion impact research conducted, but we found little evidence that a relatively greater proportion of recent studies are being conducted over multiple spatial or temporal scales. In fact, we found only 20 studies that have been conducted on plant invasion impacts across multiple spatial scales. The vast majority of observational studies have been conducted for a year or less, with most representing just a single observation event, and experimental addition studies were also usually brief, most often <1 year. Experimental removal studies were often relatively longer in duration but still relatively brief (<2 years). Study durations were similar among functional groups; although we might expect the impacts of longer-lived species (i.e. shrubs and trees) to be studied over longer time intervals in order to accurately assess their effects on the resident community, we did not find a strong pattern in the literature that this is the case. In general, we have a very poor understanding of how invasive–native interactions might change over long time periods (Yelenik and D'Antonio 2013) even though such changes could have significant implications for ecological interactions and management priorities. For example, native or introduced pathogens (Flory and Clay 2013) or insect enemies (Siemann *et al*. 2006; Brändle *et al*. 2008) may accumulate on the invasive species, native species may evolve in the face of competition from the invader (Callaway *et al*. 2005; Goergen *et al*. 2011) or successional processes may alter abiotic or biotic conditions such that they are less favourable for the introduced species (Meiners and Martinkovic 2002). Thus, there is a pressing need for more long-term studies on how the impacts of invasions may change over time.

Addressing Parker *et al.*'s second recommendation to synthesize existing data, there have been a number of efforts to review the possible and apparent impacts of biological invasions, and recently Pyšek *et al.* (2012) provided the most comprehensive data synthesis to date on community and ecosystem impacts in field studies. The third call from Parker *et al.* (1999) was more inclusion of modelling to broaden the generalities of empirical research. We found that although studies incorporating predictive models are increasing, there are still far fewer modelling studies than observational or experimental studies and relatively few studies include both empirical and modelling components. One potential reason for the scarceness of models in invasion impact studies may be a lack of training among ecologists in the skills necessary to construct models, leading many ecologists to balk at attempting to utilize these powerful tools for understanding invasion impacts. Likewise, for many invasive plant species, there are currently insufficient data to generate useful models. Nevertheless, we call for further advanced training at the graduate and undergraduate levels in statistics and modelling techniques and reiterate the call from Parker *et al.* (1999) for the continued collection of pertinent data and development of models to predict where and under what conditions invasive plants have the greatest impacts.

Our data show that over half of all studies have concentrated on the impacts of herbaceous graminoids and forbs and relatively few studies have evaluated effects of trees and shrubs (Fig. 4). In a recent review focussing on field studies addressing invasive plant impacts Hulme *et al.* (2013) used a list of 400 of the most invasive plant species worldwide to calculate the percentage of species from each plant functional group that were quantitatively assessed for invasion impacts. Similar to our review, they found that a relatively large proportion of field studies have focussed on herbaceous grasses and forbs and that invasive trees, shrubs and vines have been underrepresented in impact studies. These groups likely receive less attention because of logistical difficulties in research. Research on herbaceous species is usually much easier and short generation times and fast growth rates allow for rapid completion of experiments and publication of results. Experimental addition of trees and shrubs presents unique challenges to remove the invasion when the experiment is completed and may require longer-term studies to evaluate impacts. Because invasive trees and shrubs are known to have significant impacts on invaded communities (Jäger *et al*. 2009; Watling *et al*. 2011), more concentrated research efforts should be dedicated to evaluating their effects, despite the additional time and effort required for research on long-lived species.

Taken together, our key recommendations for methodologies that would increase understanding of invasive plant impacts are as follows:

- Studies should be designed to combine large-scale observations of invasions in natural areas with controlled removal or addition experiments that can help to elucidate cause and effect. Care should be taken in experimental studies to consider abiotic and biotic site conditions, to select biologically relevant life stages and densities of the invasive and co-occurring species and to prevent escape into surrounding areas.
- Observational and experimental studies should be conducted over two or more growing seasons and over multiple spatial scales when possible to aid in determining how the effects of invasions might change over time.
- Collaborative work among empirical and theoretical ecologists should be fostered to inform experimental design for generating appropriate data for modelling long-term effects and demographic processes. Newly generated and currently available data should be used in combination to model long-term effects of invasions. Additionally, data should be deposited whenever possible in an open-access location (e.g. datadryad.org) so that it is readily available for future predictive modelling efforts.
- Response variables related to impacts on ecosystem-level processes such as carbon and nitrogen cycling, hydrology and decomposition should be measured in studies evaluating invasive plant impacts. Common garden experiments may be particularly useful for this purpose.
- Additional experiments should be conducted to address the impacts of plant invasions on animals and microbes.

## Conclusions

Our review of over 400 peer-reviewed papers reveals that studies on the impacts of non-native plant invasions have risen dramatically in recent years, delivering valuable data to quantify the myriad impacts of invasions on native communities and ecosystems (Pyšek *et al*. 2012). Such data can aid in the prioritization of species for control, encourage the development of state and national policies for invasive species management and incentivize land managers to remove invasions. However, our review highlights the limited use of experimental research methodologies and combination studies and identifies areas where additional work is needed. Such efforts will require expanded funding opportunities at the federal, state and local level, but those costs may be recouped through reductions in ecological and economic impacts if highly damaging introduced species are identified and controlled early in the invasion process (Gardener *et al*. 2010). Given the unrelenting introductions of species to non-native ranges, ecologists must continue research to document the impacts of invasions using a combination of observational, experimental and modelling methods from the lab to the field.

## Sources of Funding

This work was funded in part by the US National Science Foundation (DEB 1257741).

## Contributions by the Authors

S.L.F. conceived the idea for the review and wrote the first draft of the paper; D.H. led the database development and assisted with editing and K.B.S. conducted statistical analyses and extensively edited the manuscript.

## Conflict of Interest Statement

None declared.

## Supporting Information

The following additional information is available in the online version of this article –

**Table S1.** Citation information for publications in the database.

**Figure S1.** Duration of studies grouped by approach and by functional group.

Additional Information
