# Supplementary material for: Improving methods to evaluate the impacts of plant invasions: lessons from 40 years of research
Source: AoB Plants. 2015 Mar 30;7:plv028. doi: 10.1093/aobpla/plv028 (PMC4418169; doi:10.1093/aobpla/plv028)
Supplement: Additional Information [file supp_7_plv028_index.html]

Improving methods to evaluate the impacts of plant invasions: lessons from 40 years of research — Additional Information 

# Improving methods to evaluate the impacts of plant invasions: lessons from 40 years of research

## Additional Information

Additional Information

**Files in this Data Supplement:**

- Supplementary File 1 - docx file
- Supplementary File 2 - doc file
