# Supplementary material for: Improving methods to evaluate the impacts of plant invasions: lessons from 40 years of research
Source: AoB Plants. 2015 Mar 30;7:plv028. doi: 10.1093/aobpla/plv028 (PMC4418169; doi:10.1093/aobpla/plv028)
Supplement: Additional Information [file supp_plv028_plv028supp_file1.docx]

**Supporting Information**

**
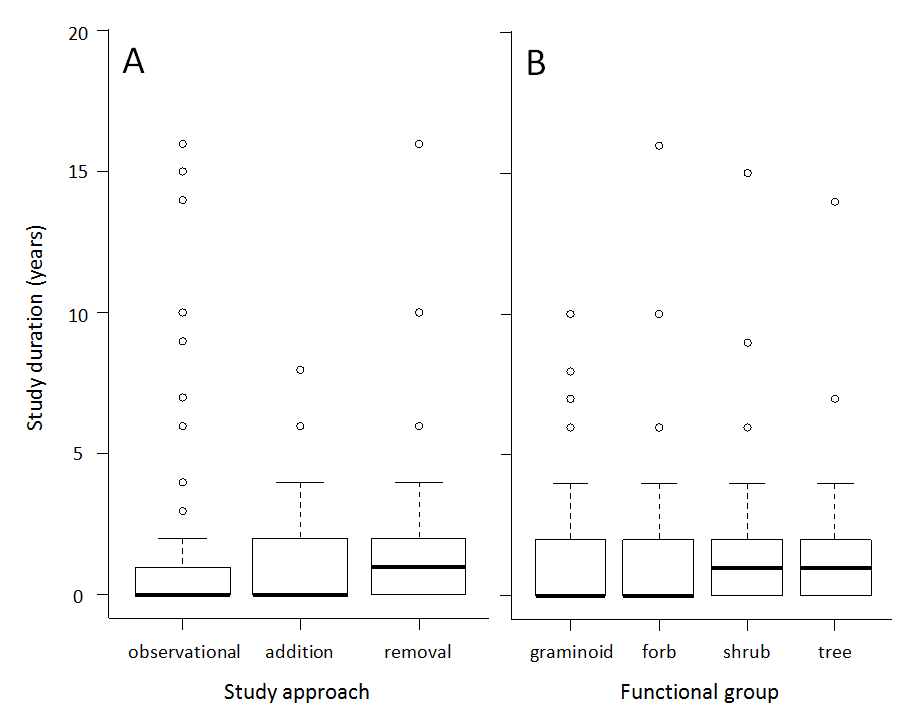
**

**Fig. 1:** Duration of studies, grouped by approach (observational, experimental addition and experimental removal) (A) and functional group (graminoid, forb, shrub, and tree) (B). Boxes represent interquartile ranges, bold lines indicate medians, whiskers represent fences, and points represent outliers.
