# Supplementary material for: Improving methods to evaluate the impacts of plant invasions: lessons from 40 years of research
Source: AoB Plants. 2015 Mar 30;7:plv028. doi: 10.1093/aobpla/plv028 (PMC4418169; doi:10.1093/aobpla/plv028)
Supplement: Additional Information [file supp_plv028_plv028supp_file2.doc]

Supporting Information. Full citation information for papers in the database.

Abhilasha, D., Quintana, N., Vivanco, J. & Joshi, J. (2008) Do allelopathic compounds in invasive *Solidago canadensis* s.l. restrain the native European flora? *Journal of Ecology*, 96, 993-1001.

Able, K.W. & Ragan, S.M. (2003) Impact of common reed, *Phragmites australis*, on essential fish habitat: Influence on reproduction, embryological development, and larval abundance of mummichog (*Fundulus heteroclitus*). *Estuaries,* 26, 40-50.

Adams, C.R. & Galatowitsch, S.M. (2006) Increasing the effectiveness of reed canary grass (*Phalaris arundineacea* L.) control in wet meadow restorations. *Restoration Ecology,* 14, 441-451.

Adams, S.N. & Engelhardt, K.A.M. (2009) Diversity declines in *Microstegium vimineum* (Japanese stiltgrass) patches. *Biological Conservation,* 142, 1003-1010.

Aguilera, A.G. (2011) The influence of soil community density on plant-soil feedbacks: An important unknown in plant invasion. *Ecological Modelling,* 222, 3413-3420.

Allan, B.F., Dutra, H.P., Goessling, L.S., Barnett, K., Chase, J.M., Marquis, R.J., Pang, G., Storch, G.A. Thach, R.E. & Orrock, J.L. (2010) Invasive honeysuckle eradication reduces tick-borne disease risk by altering host dynamics. *Proceedings of the National Academy of Sciences of the United States of America,* 107, 18523-18527.

Allen, E.B. & Knight, D.H. (1984) The effects of introduced annuals on secondary succession in sagebrush-grassland, Wyoming. *Southwestern Naturalist,* 29, 407-421.

Allison, S.D., Nielsen, C. & Hughes, R.F. (2006) Elevated enzyme activities in soils under the invasive nitrogen-fixing tree *Falcataria* *moluccana*. *Soil Biology & Biochemistry,* 38, 1537-1544.

Alvarez, M.E. & Cushman, J.H. (2002) Community-level consequences of a plant invasion: Effects on three habitats in coastal California. *Ecological Applications,* 12, 1434-1444.

Angeloni, N.L., Jankowski, K.J., Tuchman, N.C. & Kelly, J.J. (2006) Effects of an invasive cattail species (*Typha* x *glauca*) on sediment nitrogen and microbial community composition in a freshwater wetland. *Fems Microbiology Letters,* 263, 86-92.

Asner, G.P. & Beatty, S.W. (1996) Effects of an African grass invasion on Hawaiian shrubland nitrogen biogeochemistry. *Plant and Soil,* 186, 205-211.

Asner, G.P., Hughes, R.F., Vitousek, P.M., Knapp, D.E., Kennedy-Bowdoin, T., Boardman, J., Martin, R.E., Eastwood, M. & Green, R.O. (2008) Invasive plants transform the three-dimensional structure of rain forests. *Proceedings of the National Academy of Sciences of the United States of America,* 105, 4519-4523.

Asner, G.P. & Vitousek, P.M. (2005) Remote analysis of biological invasion and biogeochemical change. *Proceedings of the National Academy of Sciences of the United States of America,* 102, 4383-4386.

Atwater, D.Z., Bauer, C.M. & Callaway, R.M. (2011) Indirect positive effects ameliorate strong negative effects of *Euphorbia esula* on a native plant. *Plant Ecology,* 212, 1655-1662.

Atwood, T.B., Wiegner, T.N., Turner, J.P. & MacKenzie, R.A. (2010) Potential effects of an invasive nitrogen-fixing tree on a Hawaiian stream food web. *Pacific Science,* 64, 367-379.

Badano, E.I. & Pugnaire, F.I. (2004) Invasion of *Agave* species (Agavaceae) in south-east Spain: invader demographic parameters and impacts on native species. *Diversity and Distributions,* 10, 493-500.

Baer, S.G., Church, J.M., Williard, K.W.J. & Groninger, J.W. (2006) Changes in intrasystem N cycling from N-2-fixing shrub encroachment in grassland: multiple positive feedbacks. *Agriculture Ecosystems & Environment,* 115, 174-182.

Bahm, M.A., Barnes, T.G. & Jensen, K.C. (2011) Restoring native plant communities in smooth brome (*Bromus inermis*)-dominated grasslands. *Invasive Plant Science and Management,* 4, 239-250.

Baider, C. & Florens, F.B.V. (2011) Control of invasive alien weeds averts imminent plant extinction. *Biological Invasions,* 13, 2641-2646.

Bakker, J. & Wilson, S. (2001) Competitive abilities of introduced and native grasses. *Plant Ecology*, 157, 117-125.

Barney, J.N., Sparks, J.P., Greenberg, J., Whitlow, T.H. & Guenther, A. (2009) Biogenic volatile organic compounds from an invasive species: impacts on plant-plant interactions. *Plant Ecology,* 203, 195-205.

Barto, E.K. & Cipollini, D. (2009) Garlic mustard (*Alliaria petiolata*) removal method affects native establishment. *Invasive Plant Science and Management,* 2, 230-236.

Bartomeus, I., Vilà, M. & Santamaría, L. (2008) Contrasting effects of invasive plants in plant-pollinator networks. *Oecologia,* 155, 761-770.

Bartomeus, I., Vilà, M., Steffan-Dewenter, I. (2010) Combined effects of *Impatiens glandulifera* invasion and landscape structure on native plant pollination. *Journal of Ecology,* 98, 440-450.

Bates, J.D., Davies, K.W. & Sharp, R.N. (2011) Shrub-steppe early succession following juniper cutting and prescribed fire. *Environmental Management,* 47, 468-481.

Batten, K.M., Scow, K.M. & Espeland, E.K. (2008) Soil microbial community associated with an invasive grass differentially impacts native plant performance. *Microbial Ecology,* 55, 220-228.

Batten, K.M., Six, J., Scow, K.M. & Rillig, M.C. (2005) Plant invasion of native grassland on serpentine soils has no major effects upon selected physical and biological properties. *Soil Biology & Biochemistry,* 37, 2277-2282.

Baughman, C., Forbis, T.A. & Provencher, L. (2010) Response of two sagebrush sites to low-disturbance, mechanical removal of piñyon and juniper. *Invasive Plant Science and Management,* 3, 122-129.

Belnap, J. & Phillips, S.L. (2001) Soil biota in an ungrazed grassland: Response to annual grass (*Bromus tectorum*) invasion. *Ecological Applications,* 11, 1261-1275.

Belnap, J., Phillips, S.L., Sherrod, S.K. & Moldenke, A. (2005) Soil biota can change after exotic plant invasion: Does this affect ecosystem processes? *Ecology,* 86, 3007-3017.

Belnap, J., Phillips, S.L. & Troxler, T. (2006) Soil lichen and moss cover and species richness can be highly dynamic: The effects of invasion by the annual exotic grass *Bromus tectorum*, precipitation, and temperature on biological soil crusts in SE Utah. *Applied Soil Ecology,* 32, 63-76.

Belote, R.T., Makarick, L.J., Kearsley, M.J.C. & Lauver, C.L. (2010) Tamarisk removal in Grand Canyon National Park: Changing the native-non-native relationship as a restoration goal. *Ecological Restoration,* 28, 449-459.

Berry, Z.C., Wevill, K. & Curran, T.J. (2011) The invasive weed *Lantana camara* increases fire risk in dry rainforest by altering fuel beds. *Weed Research,* 51, 525-533.

Biggerstaff, M.S. & Beck, C.W. (2007) Effects of English ivy (*Hedera helix*) on seed bank formation and germination. *American Midland Naturalist,* 157, 250-257.

Biggerstaff, M.S. & Beck, C.W. (2007) Effects of method of English ivy removal and seed addition on regeneration of vegetation in a southeastern piedmont forest. *American Midland Naturalist,* 158, 206-220.

Blank, R.R. (2008) Biogeochemistry of plant invasion: A case study with downy brome (*Bromus tectorum*). *Invasive Plant Science and Management,* 1, 226-239.

Blank, R.R. & Young, J.A. (2002) Influence of the exotic invasive crucifer, *Lepidium latifolium*, on soil properties and elemental cycling. *Soil Science,* 167, 821-829.

Boswell, C.C. & Espie, P.R. (1998) Uptake of moisture and nutrients by *Hieracium pilosella* and effects on soil in a dry sub-humid grassland. *New Zealand Journal of Agricultural Research,* 41, 251-261.

Boyer, K.E. & Burdick, A.P. (2010) Control of *Lepidium latifolium* (perennial pepperweed) and recovery of native plants in tidal marshes of the San Francisco Estuary. *Wetlands Ecology and Management,* 18, 731-743.

Brabec, J. & Pyšek P. (2000). Establishment and survival of three invasive taxa of the genus *Reynoutria* (Polygonaceae) in mesic mown meadows: A field experimental study. *Folia Geobotanica,* 35, 27-42.

Bradford, M.A., Schumacher, H.B., Catovsky, S., Eggers, T., Newingtion, J.E. & Tordoff, G.M. (2007) Impacts of invasive plant species on riparian plant assemblages: interactions with elevated atmospheric carbon dioxide and nitrogen deposition. *Oecologia,* 152, 791-803.

Bradley, B.A., Houghton, R.A., Mustard, J.F. & Hamburg, S.P. (2006) Invasive grass reduces aboveground carbon stocks in shrublands of the Western US. *Global Change Biology,* 12, 1815-1822.

Braithwaite, R.W., Lonsdale, W.M. & Estbergs, J.A. (1989) Alien vegetation and native biota in tropical Australia - the impact of *Mimosa pigra*. *Biological Conservation,* 48, 189-210.

Brandon, A.L., Gibson, D.J. & Middleton, B.A. (2004) Mechanisms for dominance in an early successional old field by the invasive non-native *Lespedeza cuneata* (Dum. Cours.) G. Don. *Biological Invasions,* 6, 483-493.

Brockway, D.G., Gatewood, R.G. & Paris, R.B. (2002) Restoring grassland savannas from degraded piñyon-juniper woodlands: effects of mechanical overstory reduction and slash treatment alternatives. *Journal of Environmental Management,* 64, 179-197.

Brooks, M.L. (2000) Competition between alien annual grasses and native annual plants in the Mojave Desert. *American Midland Naturalist,* 144, 92-108.

Brown, B.J. & Mitchell, R.J. (2001) Competition for pollination: effects of pollen of an invasive plant on seed set of a native congener. *Oecologia,* 129, 43-49.

Brown, B.J., Mitchell, R.J. & Graham, S.A. (2002) Competition for pollination between an invasive species (purple loosestrife) and a native congener. *Ecology,* 83, 2328-2336.

Brown, C.J., Blossey, B., Maerz, J.C. & Joule, S.J. (2006) Invasive plant and experimental venue affect tadpole performance. *Biological Invasions,* 8, 327-338.

Brown, K.A., Scatena, F.N. & Gurevitch, J. (2006) Effects of an invasive tree on community structure and diversity in a tropical forest in Puerto Rico. *Forest Ecology and Management,* 226, 145-152.

Broz, A.K., Manter, D.K. & Vivanco, J.M. (2007) Soil fungal abundance and diversity: another victim of the invasive plant *Centaurea maculosa*. *The* *ISME Journal* 1, 763-765.

Brudvig, L.A. & Evans, C.W. (2006) Competitive effects of native and exotic shrubs on *Quercus alba* seedlings. *Northeastern Naturalist,* 13, 259-268.

Burch, P.L. & Zedaker, S.M. (2003) Removing the invasive tree *Ailanthus altissima* and restoring natural cover. *Journal of Arboriculture,* 29, 18-24.

Burke, D.J. (2008) Effects of *Alliaria petiolata* (garlic mustard; Brassicaceae) on mycorrhizal colonization and community structure in three herbaceous plants in a mixed deciduous forest. *American Journal of Botany,* 95, 1416-1425.

Butler, D.W. & Fairfax, R.J. (2003) Buffel grass and fire in a Gidgee and Brigalow woodland: A case study from central Queensland. *Ecological Management & Restoration,* 4, 120-125.

Caldwell, B.A. (2006) Effects of invasive scotch broom on soil properties in a Pacific coastal prairie soil. *Applied Soil Ecology,* 32, 149-152.

Cameron, G.N. & Spencer, S.R. (1989) Rapid leaf decay and nutrient release in a Chinese tallow forest. *Oecologia,* 80, 222-228.

Cannon, J.P., Allen, E.B., Allen, M.F., Dudley, L.M. & Jurinak, J.J. (1995) The effects of oxalates produced by *Salsola tragus* on the phosphorus nutrition of *Stipa pulchra*. *Oecologia,* 102, 265-272.

Carrillo-Gavilan, M.A., Lalagüe, H. & Vilà, M. (2010) Comparing seed removal of 16 pine species differing in invasiveness. *Biological Invasions,* 12, 2233-2242.

Castro-Díez, P., González-Muñoz, N., Alonso, A., Gallardo, A. & Poorter, L. (2009) Effects of exotic invasive trees on nitrogen cycling: a case study in Central Spain. *Biological Invasions,* 11, 1973-1986.

Chabrerie, O., Loinard, J., Perrin, S., Saguez, R. & Decocq, G. (2010) Impact of *Prunus serotina* invasion on understory functional diversity in a European temperate forest. *Biological Invasions,* 12, 1891-1907.

Chapuis-Lardy, L., Vanderhoeven S., Dassonville, N., Koutika, L.S. & Meerts, P. (2006) Effect of the exotic invasive plant *Solidago gigantea* on soil phosphorus status. *Biology and Fertility of Soils,* 42, 481-489.

Chen, B.M., Peng, S.L. & Ni, G.Y. (2009) Effects of the invasive plant *Mikania micrantha* H.B.K. on soil nitrogen availability through allelopathy in South China. *Biological Invasions,* 11, 1291-1299.

Chen, H., Li, B., Fang, C., Chen, J. & Wu, J. (2007) Exotic plant influences soil nematode communities through litter input. *Soil Biology & Biochemistry,* 39, 1782-1793.

Chen, Z., Li, B., Zhong, Y. & Chen, J. (2004) Local competitive effects of introduced *Spartina alterniflora* on *Scirpus mariqueter* at Dongtan of Chongming Island, the Yangtze River estuary and their potential ecological consequences. *Hydrobiologia,* 528, 99-106.

Chittka, L. & Schurkens, S. (2001) Successful invasion of a floral market - An exotic Asian plant has moved in on Europe's river-banks by bribing pollinators. *Nature*, 411, 653-653.

Chmura, D. & Sierka, E. (2006) Relation between invasive plant and species richness of forest floor vegetation: A study of *Impatiens parviflora* DC. *Polish Journal of Ecology,* 54, 417-428.

Christian, J. M. & Wilson, S. D. (1999) Long-term ecosystem impacts of an introduced grass in the northern Great Plains. *Ecology,* 80, 2397-2407.

Cipollini, D. & Darning, M. (2008) Direct and indirect effects of conditioned soils and tissue extracts of the invasive shrub, *Lonicera maackii*, on target plant performance. *Castanea,* 73, 166-176.

Cipollini, K., Ames, E. & Cipollini, D. (2009) Amur honeysuckle (*Lonicera maackii*) management method impacts restoration of understory plants in the presence of white-tailed deer (*Odocoileus virginiana*). *Invasive Plant Science and Management,* 2, 45-54.

Cipollini, K.A., McClain, G.Y. & Cipollini, D. (2008) Separating above- and belowground effects of *Alliaria petiolata* and *Lonicera maackii* on the performance of *Impatiens capensis*. *American Midland Naturalist,* 160, 117-128.

Cline, J.F., Uresk, D.W. & Rickard, W.H. (1977) Comparison of soil water used by a sagebrush bunchgrass and a cheatgrass community. *Journal of Range Management,* 30, 199-201.

Collier, M.H., Vankat, J.L. & Hughes, M.R. (2002) Diminished plant richness and abundance below *Lonicera maackii*, an invasive shrub. *American Midland Naturalist,* 147, 60-71.

Combs, J.K., Reichard, S.H., Groom, M.J., Wilderman, D.L. & Camp, P.A. (2011) Invasive competitor and native seed predators contribute to rarity of the narrow endemic *Astragalus sinuatus* Piper. *Ecological Applications,* 21, 2498-2509.

Condon, L., Weisberg, P.J. & Chambers, J.C. (2011) Abiotic and biotic influences on *Bromus tectorum* invasion and *Artemisia tridentata* recovery after fire. *International Journal of Wildland Fire,* 20, 597-604.

Conner, J.K. & Rush, S. (1996) Effects of flower size and number on pollinator visitation to wild radish, *Raphanus raphanistrum*. *Oecologia,* 105, 509-516.

Cordell, S. & Sandquist, D. R. (2008) The impact of an invasive African bunchgrass (*Pennisetum setaceum*) on water availability and productivity of canopy trees within a tropical dry forest in Hawaii. *Functional Ecology,* 22, 1008-1017.

Coultrap, D.E., Fulgham, K.O., Lancaster, D.L. Gustafson, J., Lile, D.F. & George, M.R. (2008) Relationships between western juniper (*Juniperus occidentalis*) and understory vegetation. *Invasive Plant Science and Management,* 1, 3-11.

Crimmins, T.M. & McPherson, G. R. (2008) Vegetation and seedbank response to *Eragrostis lehmanniana* removal in semi-desert communities. *Weed Research,* 48, 542-551.

Cronin, J.T. & Haynes, K. J. (2004) An invasive plant promotes unstable host-parasitoid patch dynamics. *Ecology,* 85, 2772-2782.

Cushman, J. H. & Gaffney, K.A. (2010) Community-level consequences of invasion: impacts of exotic clonal plants on riparian vegetation. *Biological Invasions,* 12, 2765-2776.

D’Antonio, C.M. & Mahall, B.E. (1991) Root profiles and competition between the invasive exotic perennial *Carpobrotus edulis* and two native shrub species in California coastal shrub. *American Journal of Botany,* 78, 885-894.

D'Antonio, C.M., Hughes, R.F., Mack, M., Hitchcock, D. & Vitousek, P.M. (1998) The response of native species to removal of invasive exotic grasses in a seasonally dry Hawaiian woodland. *Journal of Vegetation Science,* 9, 699-712.

Daehler, C.C. & Carino, D.A. (1998) Recent replacement of native pili grass (*Heteropogon contortus*) by invasive African grasses in the Hawaiian Islands. *Pacific Science,* 52, 220-227.

Dangremond, E.M., Pardini, E.A. & Knight, T.M. (2010) Apparent competition with an invasive plant hastens the extinction of an endangered lupine. *Ecology,* 91, 2261-2271.

Darning, M. & Cipollini, D. (2006) Leaf and root extracts of the invasive shrub, *Lonicera maackii*, inhibit seed germination of three herbs with no autotoxic effects. *Plant Ecology*, 184, 287-296.

Dassonville, N., Guillaumaud, N., Piola, F., Meerts, P. & Poly, F. (2011) Niche construction by the invasive Asian knotweeds (species complex *Fallopia*): impact on activity, abundance and community structure of denitrifiers and nitrifiers. *Biological Invasions,* 13, 1115-1133.

Dassonville, N., Vanderhoeven, S., Gruber, W. & Meerts, P. (2007) Invasion by *Fallopia japonica* increases topsoil mineral nutrient concentrations. *Ecoscience,* 14, 230-240.

Davalos, A. & Blossey, B. (2004) Influence of the invasive herb garlic mustard (*Alliaria petiolata*) on ground beetle (Coleoptera : Carabidae) assemblages. *Environmental Entomology,* 33, 564-576.

Davies, K.W. (2011) Plant community diversity and native plant abundance decline with increasing abundance of an exotic annual grass. *Oecologia,* 167, 481-491.

de Groot, M., Kleijn, D. & Jogan, N. (2007) Species groups occupying different trophic levels respond differently to the invasion of semi-natural vegetation by *Solidago canadensis*. *Biological Conservation,* 136, 612-617.

de Rouw, A. (1991) The invasion of *Chromolaena odorata* (L) King and Robinson (ex *Eupatorium odoratum*), and competition with the native flora in a rain forest zone southwest Ivory Coast. *Journal of Biogeography*, 18, 13-23.

Dean, W.R.J., Anderson, M.D., Milton, S.J. & Anderson, T.A. (2002) Avian assemblages in native *Acacia* and alien *Prosopis* drainage line woodland in the Kalahari, South Africa. *Journal of Arid Environments,* 51, 1-19.

DeFalco, L.A., Fernandez, G.C.J. & Nowak, R.S. (2007) Variation in the establishment of a non-native annual grass influences competitive interactions with Mojave Desert perennials. *Biological Invasions,* 9, 293-307.

Dehlin, H., Peltzer, D.A., Allison, V.J., Yeates, G.W., Nilsson, M.C. & Wardle, D.A. (2008) Tree seedling performance and below-ground properties in stands of invasive and native tree species. *New Zealand Journal of Ecology,* 32, 67-79.

DeMeester, J. E. & Richter, D. d. (2010) Restoring restoration: removal of the invasive plant *Microstegium vimineum* from a North Carolina wetland. *Biological Invasions,* 12, 781-793.

Denoth, M. & Myers, J.H. (2007) Competition between *Lythrum salicaria* and a rare species: combining evidence from experiments and long-term monitoring. *Plant Ecology,* 191, 153-161.

Dietzsch, A.C., Stanley, D.A. & Stout, J.C. (2011) Relative abundance of an invasive alien plant affects native pollination processes. *Oecologia,* 167, 469-479.

Dillemuth, F.P., Rietschier, E.A. & Cronin, J.T. (2009) Patch dynamics of a native grass in relation to the spread of invasive smooth brome (*Bromus inermis*). *Biological Invasions,* 11, 1381-1391.

Dillenburg, L.R., Whigham, D.F., Teramura, A.H., & Forseth, I.N. (1993) Effects of belowground and aboveground competition from the vines *Lonicera japonica* and *Parthenocissus quinquefolia* on the growth of the tree host *Liquidambar styraciflua*. *Oecologia*, 93, 48-54.

DiTomaso, J. M., Drewitz, J. J. & Kyser, G.B. (2008) Jubatagrass (*Cortaderia jubata*) control using chemical and mechanical methods. *Invasive Plant Science and Management,* 1, 82-90.

Djurdjević, L., Mitrović, M., Gajić, G., Jarić, S., Kostić, O., Oberan, L. & Pavlović, P. (2011) An allelopathic investigation of the domination of the introduced invasive *Conyza canadensis* L. *Flora,* 206, 921-927.

Domènech, R., Vilà, M., Gesti, J., Serrasolses, I. (2006) Neighbourhood association of *Cortaderia selloana* invasion, soil properties and plant community structure in Mediterranean coastal grasslands. *Acta Oecologica,* 29, 171-177.

Dormaar, J.F., Naeth, M.A., Willms, W.D. & Chanasyk, D.S. (1995) Effect of native prairie crested wheatgrass (*Agropyron cristatum* (L) Gaertn.) and Russian wildrye (*Elymus junceus* Fisch.) on soil chemical properties. *Journal of Range Management,* 48, 258-263.

Douglas, M.M. & O'Connor, R.A. (2003) Effects of the exotic macrophyte, para grass (*Urochloa mutica*), on benthic and epiphytic macroinvertebrates of a tropical floodplain. *Freshwater Biology,* 48, 962-971.

Douglas, M.M., Setterfield, S.A., Rossiter, N., Barratt, J. & Hutley, L.B. (2004) Effects of mission grass (*Pennisetum polystachion* (L.) Schult.) invasion on fuel loads and nitrogen availability in a northern Australia tropical savanna. *Proceedings of the 14th Australian weeds conference* (ed. by B.M. Sindel & S. B. Johnson), pp. 179–81. Weed Society of New South Wales, Sydney.

Drenovsky, R.E. & Batten, K. M. (2007). Invasion by *Aegilops triuncialis* (barb goatgrass) slows carbon and nutrient cycling in a serpentine grassland. *Biological Invasions,* 9, 107-116.

Dunbar, K.R. & Facelli, J. M. (1999) The impact of a novel invasive species, *Orbea variegata* (African carrion flower), on the chenopod shrublands of South Australia. *Journal of Arid Environments,* 41, 37-48.

Durst, S.L., Theimer, T.C., Paxton, E.H. & Sogge, M.K. (2008) Temporal variation in the arthropod community of desert riparian habitats with varying amounts of saltcedar (*Tamarix ramosissima*). *Journal of Arid Environments,* 72, 1644-1653.

Ehrenfeld, J.G., Kourtev, P. & Huang, W. (2001) Changes in soil functions following invasions of exotic understory plants in deciduous forests. *Ecological Applications,* 11, 1287-1300.

Ellingson, A.R. & Andersen, D.C. (2002) Spatial correlations of *Diceroprocta apache* and its host plants: evidence for a negative impact from *Tamarix* invasion. *Ecological Entomology,* 27, 16-24.

Ellis, L.M., Crawford, C.S., Molles, M.C. (1998) Comparison of litter dynamics in native and exotic riparian vegetation along the Middle Rio Grande of central New Mexico, USA. *Journal of Arid Environments,* 38, 283-296.

Enloe, S.F., DiTomaso, J.M., Orloff, S.B. & Drake, D.J. (2004) Soil water dynamics differ among rangeland plant communities dominated by yellow starthistle (*Centaurea solstitialis*), annual grasses, or perennial grasses. *Weed Science,* 52, 929-935.

Ens, E.J. & French, K. (2008) Exotic woody invader limits the recruitment of three indigenous plant species. *Biological Conservation,* 141, 590-595.

Equihua, M. & Usher, M.B. (1993) Impact of carpets of the invasive moss *Campylopus introflexus* on *Calluna vulgaris* regeneration. *Journal of Ecology,* 81, 359-365.

Ernst, C. M. & Cappuccino, N. (2005) The effect of an invasive alien vine, *Vincetoxicum rossicum* (Asclepiadaceae), on arthropod populations in Ontario old fields. *Biological Invasions,* 7, 417-425.

Ervin, G.N. & Wetzel, R.G. (2002) Influence of a dominant macrophyte, *Juncus effusus*, on wetland plant species richness, diversity, and community composition. *Oecologia,* 130, 626-636.

Evans, R.D., Rimer, R., Sperry, L. & Belnap, J. (2001) Exotic plant invasion alters nitrogen dynamics in an arid grassland. *Ecological Applications,* 11, 1301-1310.

Farnsworth, E.J. & Meyerson, L.A. (1999) Species composition and inter-annual dynamics of a freshwater tidal plant community following removal of the invasive grass, *Phragmites australis*. *Biological Invasions,* 1, 115-127.

Feher, A. & Koncekova, L. (2009) Evaluation of mechanical regulation of invasive *Helianthus tuberosus* populations in agricultural landscape. *Journal of Central European Agriculture,* 10, 245-250.

Fickbohm, S.S. & Zhu, W.X. (2006) Exotic purple loosestrife invasion of native cattail freshwater wetlands: Effects on organic matter distribution and soil nitrogen cycling. *Applied Soil Ecology,* 32, 123-131.

Finch, O.D. & Szumelda, A. (2007) Introduction of Douglas fir (*Pseudotsuga menziesii* (Mirb.) Franco) into Western Europe: Epigaeic arthropods in intermediate-aged pure stands in northwestern Germany. *Forest Ecology and Management,* 242, 260-272.

Findlay, S., Groffman, P. & Dye, S. (2003) Effects of *Phragmites australis* removal on marsh nutrient cycling. *Wetlands Ecology and Management,* 11, 157-165.

Fischer, L. K., von der Lippe, M. & Kowarik, I. (2009) Tree invasion in managed tropical forests facilitates endemic species. *Journal of Biogeography,* 36, 2251-2263.

Fisher, J.L., Veneklaas, E.J., Lambers, H. & Loneragan, W.A. (2006) Enhanced soil and leaf nutrient status of a Western Australian *Banksia* woodland community invaded by *Ehrharta calycina* and *Pelargonium capitatum*. *Plant and Soil,* 284, 253-264.

Fleishman, E., Mac Nally, R. & Murphy, D.D. (2005) Relationships among non-native plants, diversity of plants and butterflies, and adequacy of spatial sampling. *Biological Journal of the Linnean Society,* 85, 157-166.

Flory, S.L. & Clay, K. (2009) Invasive plant removal method determines native plant community responses. *Journal of Applied Ecology,* 46, 434-442.

Flory, S.L. & Clay, K. (2010) Non-native grass invasion suppresses forest succession. *Oecologia,* 164, 1029-1038.

Flory, S.L. & Clay, K. (2010) Non-native grass invasion alters native plant composition in experimental communities. *Biological Invasions,* 12, 1285-1294.

Flory, S.L. & Lewis, J. (2009) Nonchemical methods for managing Japanese stiltgrass (*Microstegium vimineum*). *Invasive Plant Science and Management,* 2, 301-308.

Frappier, B., Eckert, R.T. & Lee, T.D. (2003) Potential impacts of the invasive exotic shrub *Rhamnus frangula* L. (glossy buckthorn) on forests of southern New Hampshire. *Northeastern Naturalist,* 10, 277-296.

Frappier, B., Eckert, R.T. & Lee, T.D. (2004) Experimental removal of the non-indigenous shrub *Rhamnus frangula* (glossy buckthorn): Effects on native herbs and woody seedlings. *Northeastern Naturalist,* 11, 333-342.

Fraser, L.H. & Carlyle, C.N. (2011) Is spotted knapweed (*Centaurea stoebe* L.) patch size related to the effect on soil and vegetation properties? *Plant Ecology,* 212, 975-983.

Fraterrigo, J.M., Strickland, M.S., Keiser, A.D. & Bradford, M.A. (2011) Nitrogen uptake and preference in a forest understory following invasion by an exotic grass. *Oecologia,* 167, 781-791.

Freifelder, R.R., Vitousek, P.M. & D’Antonio, C.M. (1998) Microclimate change and effect on fire following forest-grass conversion in seasonally dry tropical woodland. *Biotropica,* 30, 286-297.

French, K. & Major, R.E. (2001) Effect of an exotic *Acacia* (Fabaceae) on ant assemblages in South African fynbos. *Austral Ecology,* 26, 303-310.

French, K. & Zubovic, A. (1997) Effect of the weed *Chrysanthemoides monilifera* (bitou bush) on bird communities. *Wildlife Research,* 24, 727-735.

Funk, J. L. (2005) *Hedychium gardnerianum* invasion into Hawaiian montane rainforest: interactions among litter quality, decomposition rate, and soil nitrogen availability. *Biogeochemistry,* 76, 441-451.

Galbraith-Kent, S.L. & Handel, S.N. (2008) Invasive *Acer platanoides* inhibits native sapling growth in forest understorey communities. *Journal of Ecology,* 96, 293-302.

Gentle, C.B. & Duggin J.A. (1997) Allelopathy as a competitive strategy in persistent thickets of *Lantana camara* L. in three Australian forest communities. *Plant Ecology,* 132, 85-95.

Ghazoul, J. (2004) Alien abduction: Disruption of native plant-pollinator interactions by invasive species. *Biotropica,* 36, 156-164.

Giantomasi, A., Tecco, P.A., Funes, G., Gurvich, D.E. & Cabido, M. (2008) Canopy effects of the invasive shrub *Pyracantha angustifolia* on seed bank composition, richness and density in a montane shrubland (Cordoba, Argentina). *Austral Ecology,* 33, 68-77.

Goergen, E. & Daehler, C.C. (2001) Inflorescence damage by insects and fungi in native pili grass (*Heteropogon contortus*) versus alien fountain grass (*Pennisetum setaceum*) in Hawai'i. *Pacific Science,* 55, 129-136.

Gogo, S., Laggoun-Défarge, F., Delarue, F. & Lottier, N. (2011) Invasion of a *Sphagnum*-peatland by *Betula* spp and *Molinia caerulea* impacts organic matter biochemistry. Implications for carbon and nutrient cycling. *Biogeochemistry,* 106, 53-69.

Going, B.M. & Dudley, T.L. (2008) Invasive riparian plant litter alters aquatic insect growth. *Biological Invasions,* 10, 1041-1051.

Gómez-Aparicio, L. & Canham, C.D. (2008) Neighborhood models of the effects of invasive tree species on ecosystem processes. *Ecological Monographs,* 78, 69-86.

Gomez-Aparicio, L., Canham, C.D. & Martin, P.H. (2008) Neighbourhood models of the effects of the invasive *Acer platanoides* on tree seedling dynamics: linking impacts on communities and ecosystems. *Journal of Ecology,* 96, 78-90.

Gonzalez, C.L. & Dodd, J.D. (1979) Production reponses of native and introduced grasses to mechanical brush manipulation, seeding, and fertilization. *Journal of Range Management,* 32, 305-309.

Gould, A.M.A. & Gorchov, D.L. (2000) Effects of the exotic invasive shrub *Lonicera maackii* on the survival and fecundity of three species of native annuals. *American Midland Naturalist,* 144, 36-50.

Grant, D.W., Peters, D.P.C., Beck, G.K. & Fraleigh, H.D. (2003) Influence of an exotic species, *Acroptilon repens* (L.) DC. on seedling emergence and growth of native grasses. *Plant Ecology,* 166, 157-166.

Gratton, C. & Denno, R.F. (2005) Restoration of arthropod assemblages in a *Spartina* salt marsh following removal of the invasive plant *Phragmites australis*. *Restoration Ecology,* 13, 358-372.

Gratton, C. & Denno, R.F. (2006) Arthropod food web restoration following removal of an invasive wetland plant. *Ecological Applications,* 16, 622-631.

Green, E.K. & Galatowitsch, S.M. (2001) Differences in wetland plant community establishment with additions of nitrate-N and invasive species (*Phalaris arundinacea* and *Typha* x *glauca*). *Canadian Journal of Botany,* 79, 170-178.

Greenwood, H., O'Dowd, D.J. & Lake, P.S. (2004) Willow (*Salix* x *rubens*) invasion of the riparian zone in south-eastern Australia: reduced abundance and altered composition of terrestrial arthropods. *Diversity and Distributions,* 10, 485-492.

Gremmen, N.J.M., Chown, S.L. & Marshall, D.J. (1998) Impact of the introduced grass *Agrostis stolonifera* on vegetation and soil fauna communities at Marion Island, sub-Antarctic. *Biological Conservation,* 85, 223-231.

Hacker, S.D. & Dethier M.N. (2006) Community modification by a grass invader has differing impacts for marine habitats. *Oikos,* 113, 279-286.

Haferkamp, M.R., Volesky, J.D., Borman, M.M., Heitschmidt, R.K. & Currie, P.O. (1993) Effects of mechanical treatments and climatic factors on the productivity of northern Great Plains rangelands. *Journal of Range Management,* 46, 346-350.

Hager, H. A. (2004) Competitive effect versus competitive response of invasive and native wetland plant species. *Oecologia,* 139, 140-149.

Hall, S.J. & Asner, G.P. (2007) Biological invasion alters regional nitrogen-oxide emissions from tropical rainforests. *Global Change Biology,* 13, 2143-2160.

Hanula, J.L., Horn, S. & Taylor, J.W. (2009) Chinese privet (*Ligustrum sinense*) removal and its effect on native plant communities of riparian forests. *Invasive Plant Science and Management,* 2, 292-300.

Harcombe, P.A., Cameron, G.N. & Glumac, E.G. (1993) Above-ground net primary productivity in adjacent grassland and woodland on the coastal prairie of Texas, USA. *Journal of Vegetation Science,* 4, 521-530.

Hargraves, P.E. (2008) Allelopathy at the land/sea interface: Microalgae and Brazilian pepper. *Marine Environmental Research,* 66, 553-555.

Harms, R.S. & Hiebert, R.D. (2006) Vegetation response following invasive tamarisk (*Tamarix* spp.) removal and implications for riparian restoration. *Restoration Ecology,* 14, 461-472.

Harnden, J., MacDougall, A.S. & Sikes, B.A. (2011) Field-based effects of allelopathy in invaded tallgrass prairie. *Botany-Botanique,* 89, 227-234.

Harris, W. & Fan, J. (1996) The role of fertiliser in the invasion of South Island high country by hawkweeds. *Proceedings of the New Zealand Grassland Association,* 58, 205-210.

Harrison, S., Grace, J.B., Davies, K.E., Safford, H.D. & Viers, J.H. (2006) Invasion in a diversity hotspot: Exotic cover and native richness in the Californian serpentine flora. *Ecology,* 87, 695-703.

Hartman, K.M. & McCarthy, B.C. (2004) Restoration of a forest understory after the removal of an invasive shrub, Amur honeysuckle (*Lonicera maackii*). *Restoration Ecology,* 12, 154-165.

Hartman, K.M. & McCarthy, B.C. (2007). A dendro-ecological study of forest overstorey productivity following the invasion of the non-indigenous shrub *Lonicera maackii*. *Applied Vegetation Science,* 10, 3-14.

Haubensak, K.A. & Parker, I.M. (2004) Soil changes accompanying invasion of the exotic shrub *Cytisus scoparius* in glacial outwash prairies of western Washington USA. *Plant Ecology,* 175, 71-79.

Hawkes, C.V., Belnap, J., D’Antonio, C. & Firestone, M.K. (2006) Arbuscular mycorrhizal assemblages in native plant roots change in the presence of invasive exotic grasses. *Plant and Soil,* 281, 369-380.

Hedge, P. & Kriwoken, L.K. (2000) Evidence for effects of *Spartina anglica* invasion on benthic macrofauna in Little Swanport estuary, Tasmania. *Austral Ecology,* 25, 150-159.

Hejda, M. &, P. (2006). What is the impact of *Impatiens glandulifera* on species diversity of invaded riparian vegetation? *Biological Conservation,* 132, 143-152.

Hejda, M., Pyšek, P. & Jarošik, V. (2009) Impact of invasive plants on the species richness, diversity and composition of invaded communities. *Journal of Ecology,* 97, 393-403.

Heleno, R., Lacerda, I., Ramos, J.A. & Memmott, J. (2010) Evaluation of restoration effectiveness: community response to the removal of alien plants. *Ecological Applications,* 20, 1191-1203.

Henderson, D.C. & Naeth M.A. (2005) Multi-scale impacts of crested wheatgrass invasion in mixed-grass prairie. *Biological Invasions,* 7, 639-650.

Heneghan, L., Fatemi, F., Umek, L., Grady, K., Fagen, K. & Workman, M. (2006) The invasive shrub European buckthorn (*Rhamnus cathartica*, L.) alters soil properties in Midwestern US woodlands. *Applied Soil Ecology,* 32, 142-148.

Herr, C., Chapuis-Lardy, L., Dassonville, N., Vanderhoeven, S. & Meerts, P. (2007) Seasonal effect of the exotic invasive plant *Solidago gigantea* on soil pH and P fractions. *Journal of Plant Nutrition and Soil Science,* 170, 729-738.

Herrera, A.M. & Dudley, T.L. (2003) Reduction of riparian arthropod abundance and diversity as a consequence of giant reed (*Arundo donax*) invasion. *Biological Invasions,* 5, 167-177.

Hickman, J.E., Wu, S., Mickley, L.J. & Lerdau, M.T. (2010) Kudzu (*Pueraria montana*) invasion doubles emissions of nitric oxide and increases ozone pollution. *Proceedings of the National Academy of Sciences of the United States of America* 107, 10115-10119.

Hoffmann, W.A. & Haridasan, M. (2008) The invasive grass, *Melinis minutiflora*, inhibits tree regeneration in a Neotropical savanna. *Austral Ecology,* 33, 29-36.

Hoffmann, W.A., Lucatelli, V.M.P.C., Silva, F.J., Azeuedo, I.N.C., Marinho, M.D., Albuquerque, A.M.S., Lopes, A.D. & Moreira, S.P. (2004) Impact of the invasive alien grass *Melinis minutiflora* at the savanna-forest ecotone in the Brazilian Cerrado. *Diversity and Distributions,* 10, 99-103.

Holly, D.C., Ervin, G.N., Jackson, C.R., Diehl, S.V. & Kirker, G.T. (2009) Effect of an invasive grass on ambient rates of decomposition and microbial community structure: a search for causality. *Biological Invasions,* 11, 1855-1868.

Holmes, P.M. & Cowling, R.M. (1997) The effects of invasion by *Acacia saligna* on the guild structure and regeneration capabilities of South African fynbos shrublands. *Journal of Applied Ecology,* 34, 317-332.

Holmquist, J.G., Schmidt-Gengenbach, J. & Slaton, M.R. (2011) Influence of invasive palms on terrestrial arthropod assemblages in desert spring habitat. *Biological Conservation,* 144, 518-525.

Hook, P.B., Olson, B.E. & Wraith, J.M. (2004) Effects of the invasive forb *Centaurea maculosa* on grassland carbon and nitrogen pools in Montana, USA. *Ecosystems,* 7, 686-694.

Hoopes, M.F. & Hall, L.M. (2002) Edaphic factors and competition affect pattern formation and invasion in a California grassland. *Ecological Applications,* 12, 24-39.

Houston, W.A. & Duivenvoorden, L.J. (2002) Replacement of littoral native vegetation with the ponded pasture grass *Hymenachne amplexicaulis*: effects on plant, macroinvertebrate and fish biodiversity of backwaters in the Fitzroy River, Central Queensland, Australia. *Marine and Freshwater Research,* 53, 1235-1244.

Hua, C., Jian, L., Yongli, Z., Qiang, W., Xiuli, G., Yingua, W., & Renging, W. (2011) Influence of invasive plant *Coreopsis grandiflora* on functional diversity of soil microbial communities. *Journal of Environmental Biology*, 32, 567-572.

Huenneke, L.F., Hamburg, S.P., Koide, R., Mooney, H.A. & Vitousek, P.M. (1990) Effects of soil resources on plant invasion and community structure in Californian serpentine grassland. *Ecology,* 71, 478-491.

Huenneke, L.F. & Thomson, J.K. (1995) Potential interference between a threatened endemic thistle and an invasive nonnative plant. *Conservation Biology,* 9, 416-425.

Hughes, F., Vitousek, P.M. & Tunison, T. (1991) Alien grass invasion and fire in the seasonal submontane zone of Hawaii. *Ecology,* 72, 743-746.

Hughes, R.F. & Denslow, J.S. (2005) Invasion by a N-2-fixing tree alters function and structure in wet lowland forests of Hawaii. *Ecological Applications,* 15, 1615-1628.

Hughes, R.F. & Uowolo, A. (2006) Impacts of *Falcataria moluccana* invasion on decomposition in Hawaiian lowland wet forests: The importance of stand-level controls. *Ecosystems,* 9, 977-991.

Hulme, P.E. & Bremner, E.T. (2006) Assessing the impact of *Impatiens glandulifera* on riparian habitats: partitioning diversity components following species removal. *Journal of Applied Ecology,* 43, 43-50.

Iponga, D.M., Milton, S.J. & Richardson (2008) Superiority in competition for light: A crucial attribute defining the impact of the invasive alien tree *Schinus molle* (Anacardiaceae) in South African savanna. *Journal of Arid Environments,* 72, 612-623.

Jackson, J. (2005) Is there a relationship between herbaceous species richness and buffel grass (*Cenchrus ciliaris*)? *Austral Ecology,* 30, 505-517.

Jäger, H., Kowarik, I. & Tye, A. (2009) Destruction without extinction: long-term impacts of an invasive tree species on Galapagos highland vegetation. *Journal of Ecology,* 97, 1252-1263.

Jäger, H., Tye, A. & Kowarik, I. (2007) Tree invasion in naturally treeless environments: Impacts of quinine (*Cinchona pubescens*) trees on native vegetation in Galapagos. *Biological Conservation,* 140, 297-307.

Johnson, N.C. & Wedin, D.A. (1997) Soil carbon, nutrients, and mycorrhizae during conversion of dry tropical forest to grassland. *Ecological Applications,* 7, 171-182.

Jordan, N.R., Larson, D.L. & Huerd, S.C. (2008) Soil modification by invasive plants: effects on native and invasive species of mixed-grass prairies. *Biological Invasions,* 10, 177-190.

Jordan, N.R., Larson, D.L. & Huerd, S.C. (2011) Evidence of qualitative differences between soil-occupancy effects of invasive vs. native grassland plant species. *Invasive Plant Science and Management,* 4, 11-21.

Joshi, A.A., Mudappa, D. & Raman, T.R.S. (2009) Brewing trouble: coffee invasion in relation to edges and forest structure in tropical rainforest fragments of the Western Ghats, India. *Biological Invasions,* 11, 2387-2400.

Judge, C.A., Neal, J.C. & Shear, T.H. (2008) Japanese stiltgrass (*Microstegium vimineum*) management for restoration of native plant communities. *Invasive Plant Science and Management,* 1, 111-119.

Kao-Kniffin, J. & Balser, T.C. (2008) Soil fertility and the impact of exotic invasion on microbial communities in hawaiian forests. *Microbial Ecology,* 56, 55-63.

Kappes, H., Lay, R. & Topp, W. (2007) Changes in different trophic levels of litter-dwelling macrofauna associated with giant knotweed invasion. *Ecosystems,* 10, 734-744.

Keller, B.E.M. (2000) Plant diversity in *Lythrum*, *Phragmites*, and *Typha* marshes, Massachusetts, U.S.A. *Wetlands Ecology and Management,* 8, 391-401.

Kennedy, T.A., Finlay, J.C. & Hobbie, S.E. (2005) Eradication of invasive *Tamarix ramosissima* along a desert stream increases native fish density. *Ecological Applications,* 15, 2072-2083.

Kivlin, S.N. & Hawkes, C.V. (2011) Differentiating between effects of invasion and diversity: impacts of aboveground plant communities on belowground fungal communities. *New Phytologist,* 189, 526-535.

Klionsky, S.M., Amatangelo, K.L. & Waller, D.M. (2011) Above- and belowground impacts of European buckthorn (*Rhamnus cathartica*) on four native forbs. *Restoration Ecology,* 19, 728-737.

Koch, A.M., Antunes, P.M., Barto, E.K., Cipollini, D., Mummey, D.L. & Klironomos, J.N. (2011) The effects of arbuscular mycorrhizal (AM) fungal and garlic mustard introductions on native AM fungal diversity. *Biological Invasions,* 13, 1627-1639.

Kourtev, P.S., Ehrenfeld, J.G. & Häggblom, M. (2002) Exotic plant species alter the microbial community structure and function in the soil. *Ecology,* 83, 3152-3166.

Kourtev, P.S., Huang, W.Z. & Ehrenfeld, J.G. (1999) Differences in earthworm densities and nitrogen dynamics in soils under exotic and native plant species. *Biological Invasions,* 1, 237-245.

Koutika, L.S., Vanderhoeven, S., Chapuis-Lardy, L., Dassonville, N. & Meerts, P. (2007) Assessment of changes in soil organic matter after invasion by exotic plant species. *Biology and Fertility of Soils,* 44, 331-341.

Kueffer, C., Schumacher, E., Fleischmann, K., Edwards, P.J. & Dietz, H. (2007) Strong below-ground competition shapes tree regeneration in invasive *Cinnamomum verum* forests. *Journal of Ecology,* 95, 273-282.

Kurten, E.L., Snyder, C. P., Iwata, T. & Vitousek, P.M. (2008) *Morella cerifera* invasion and nitrogen cycling on a lowland Hawaiian lava flow. *Biological Invasions,* 10, 19-24.

Kwiatkowska, A.J., Spalik, K., Michalak, E., Palińska, A. & Panufnik, D. (1997) Influence of the size and density of *Carpinus betulus* on the spatial distribution and rate of deletion of forest-floor species in thermophilous oak forest. *Plant Ecology,* 129, 1-10.

Lambrinos, J.G. (2000) The impact of the invasive alien grass *Cortaderia jubata* (Lemoine) Stapf on an endangered mediterranean-type shrubland in California. *Diversity and Distributions,* 6, 217-231.

Lawrence, J.G., Colwell, A. & Sexton, O.J. (1991) The ecological impact of allelopathy in *Ailanthus altissima (*Simaroubaceae). *American Journal of Botany,* 78, 948-958.

Le Maitre, D.C., Gaertner, M., Marchante, E., Ens, E.J., Holmes, P.M., Pauchard, A., O’Farrell, P.J., Rogers, A.W., Blanchard, R., Blignaut, J. & Richardson, D.M. (2011) Impacts of invasive Australian acacias: implications for management and restoration. *Diversity and Distributions,* 17, 1015-1029.

Leary, J.K., Hue, N.V., Singleton, P.W. & Borthakur, D. (2006) The major features of an infestation by the invasive weed legume gorse (*Ulex europaeus*) on volcanic soils in Hawaii. *Biology and Fertility of Soils,* 42, 215-223.

Leichty, E.R., Carmichael, B.J. & Platt, W.J. (2011) Invasion of a Southeastern pine savanna by Japanese climbing fern. *Castanea,* 76, 293-299.

Lenz, L. & Taylor, J.A. (2001) The influence of an invasive tree species (*Myrica faya*) on the abundance of an alien insect (*Sophonia rufofascia*) in Hawai'i Volcanoes National Park. *Biological Conservation,* 102, 301-307.

Lesica, P. & DeLuca, T.H. (2004) Is tamarisk allelopathic? *Plant and Soil,* 267, 357-365.

Lesica, P. & Shelly, J.S. (1996) Competitive effects of *Centaurea maculosa* on the population dynamics of *Arabis fecunda*. *Bulletin of the Torrey Botanical Club,* 123, 111-121.

Leslie, A.J. & Spotila, J.R. (2001) Alien plant threatens Nile crocodile (*Crocodylus niloticus*) breeding in Lake St. Lucia, South Africa. *Biological Conservation,* 98, 347-355.

Levine, J.M. (2000) Species diversity and biological invasions: Relating local process to community pattern. *Science,* 288, 852-854.

Ley, R.E. & D'Antonio, C.M. (1998) Exotic grass invasion alters potential rates of N fixation in Hawaiian woodlands. *Oecologia,* 113, 179-187.

Liao, C., Luo, Y., Jiang, L., Zhou, X., Wu, X., Fang, C., Chen, J. & Li, B. (2007) Invasion of *Spartina alterniflora* enhanced ecosystem carbon and nitrogen stocks in the Yangtze Estuary, China. *Ecosystems,* 10, 1351-1361.

Lindsay, D.L., Bailey, P., Lance, R.F., Clifford, M.J., Delph, R. & Cobb, N.S. (2011) Effects of a nonnative, invasive lovegrass on *Agave palmeri* distribution, abundance, and insect pollinator communities. *Biodiversity and Conservation,* 20, 3251-3266.

Lindsay, E.A. & French, K. (2005) Litterfall and nitrogen cycling following invasion by *Chrysanthemoides monilifera* ssp *rotundata* in coastal Australia. *Journal of Applied Ecology,* 42, 556-566.

Lindsay, E.A. & French, K. (2006) The impact of the weed *Chrysanthemoides monilifera* ssp *rotundata* on coastal leaf litter invertebrates. *Biological Invasions,* 8, 177-192.

Linxiao, M., Xin, C., & Tang, J. (2005) Allelopathic effects of invasive weed *Solidago canadensis*. *Chinese Journal of Applied Ecology*, 16, 2379-2382.

Litton, C.M., Sandquist, D.R. & Cordell, S. (2006) Effects of non-native grass invasion on aboveground carbon pools and tree population structure in a tropical dry forest of Hawaii. *Forest Ecology and Management,* 231, 105-113.

Lloyd, J.D. & Martin, T. E. (2005) Reproductive success of chestnut-collared longspurs in native and exotic grassland. *Condor,* 107, 363-374.

Lopezaraiza-Mikel, M.E., Hayes, R.B., Whalley, M.R. & Memmott, J. (2007) The impact of an alien plant on a native plant-pollinator network: an experimental approach. *Ecology Letters,* 10, 539-550.

Love, J.P. & Anderson, J.T. (2009) Seasonal effects of four control methods on the invasive Morrow's honeysuckle (*Lonicera morrowii*) and initial responses of understory plants in a Southwestern Pennsylvania old field. *Restoration Ecology,* 17, 549-559.

Maerz, J.C., Blossey, B. & Nuzzo, V. (2005) Green frogs show reduced foraging success in habitats invaded by Japanese knotweed. *Biodiversity and Conservation,* 14, 2901-2911.

Maerz, J.C., Brown, C.J., Chapin, C.T. & Blossey, B. (2005) Can secondary compounds of an invasive plant affect larval amphibians? *Functional Ecology,* 19, 970-975.

Maerz, J.C., Nuzzo, V.A. & Blossey, B. (2009) Declines in woodland salamander abundance associated with non-native earthworm and plant invasions. *Conservation Biology,* 23, 975-981.

Maltez-Mouro, S., Maestre, F.T. & Freitas, H. (2010) Weak effects of the exotic invasive *Carpobrotus edulis* on the structure and composition of Portuguese sand-dune communities. *Biological Invasions,* 12, 2117-2130.

Mangla, S., Inderjit, & Callaway, R.M. (2008) Exotic invasive plant accumulates native soil pathogens which inhibit native plants. *Journal of Ecology,* 96, 58-67.

Marchante, E., Kjøller, A., Struwe, S. & Freitas, H. (2008). Invasive *Acacia longifolia* induce changes in the microbial catabolic diversity of sand dunes. *Soil Biology & Biochemistry* 40, 2563-2568.

Marchante, E., Kjøller, A., Struwe, S. & Freitas, H. (2008) Short- and long-term impacts of *Acacia longifolia* invasion on the belowground processes of a Mediterranean coastal dune ecosystem. *Applied Soil Ecology,* 40, 210-217.

Maron, J.L. & Marler, M. (2008) Field-based competitive impacts between invaders and natives at varying resource supply. *Journal of Ecology,* 96, 1187-1197.

Martin, M.R., Tipping, P.W. & Sickman, J.O. (2009) Invasion by an exotic tree alters above and belowground ecosystem components. *Biological Invasions,* 11, 1883-1894.

Martin, P.H. (1999) Norway maple (*Acer platanoides*) invasion of a natural forest stand: understory consequence and regeneration pattern. *Biological Invasions,* 1, 215-222.

Mason, T.J. & French, K. (2007) Management regimes for a plant invader differentially impact resident communities. *Biological Conservation,* 136, 246-259.

Mason, T.J. & French, K. (2008) Impacts of a woody invader vary in different vegetation communities. *Diversity and Distributions,* 14, 829-838.

Matzek, V. (2011) Superior performance and nutrient-use efficiency of invasive plants over non-invasive congeners in a resource-limited environment. *Biological Invasions,* 13, 3005-3014.

May, L. & Baldwin, L.K. (2011) Linking field based studies with greenhouse experiments: the impact of *Centaurea stoebe* (=*C. maculosa*) in British Columbia grasslands. *Biological Invasions.* 13, 919-931.

Mayer, P.M., Tunnell, S.J., Engle, D.M., Jorgensen, E.E. & Nunn, P. (2005) Invasive grass alters litter decomposition by influencing macrodetritivores. *Ecosystems,* 8, 200-209.

McCarthy, B.C. & Hanson, S.L. (1998) An assessment of the allelopathic potential of the invasive weed *Alliaria petiolata* (Brassicaceae). *Castanea,* 63, 68-73.

McGlynn, C.A. (2009) Native and invasive plant interactions in wetlands and the minimal role of invasiveness. *Biological Invasions,* 11, 1929-1939.

McGrath, D.A. & Binkley, M.A. (2009) *Microstegium vimineum* invasion changes soil chemistry and microarthropod communities in Cumberland plateau forests. *Southeastern Naturalist,* 8, 141-156.

Meffin, R., Miller, A.L., Hulme, P.E. & Duncan, R.P. (2010) Experimental introduction of the alien plant *Hieracium lepidulum* reveals no significant impact on montane plant communities in New Zealand. *Diversity and Distributions,* 16, 804-815.

Melgoza, G., Nowak, R.S. & Tausch, R.J. (1990) Soil water exploitation after fire: competition between *Bromus tectorum* (cheatgrass) and two native species. *Oecologia,* 83, 7-13.

Meyerson, L.A., Chambers, R.M. & Vogt, K.A. (1999) The effects of *Phragmites* removal on nutrient pools in a freshwater tidal marsh ecosystem. *Biological Invasions,* 1, 129-136.

Mgobozi, M.P., Somers, M.J. & Dippenaar-Schoeman, A.S. (2008) Spider responses to alien plant invasion: the effect of short- and long-term *Chromolaena odorata* invasion and management. *Journal of Applied Ecology,* 45, 1189-1197.

Miller, A.L. & Duncan, R.P. (2004 The impact of exotic weed competition on a rare New Zealand outcrop herb, *Pachycladon cheesemanii* (Brassicaceae). *New Zealand Journal of Ecology,* 28, 113-124.

Mills, J.E., Reinartz, J.A., Meyer, G.A. & Young, E.B. (2009) Exotic shrub invasion in an undisturbed wetland has little community-level effect over a 15-year period. *Biological Invasions,* 11, 1803-1820.

Minchinton, T.E., Simpson, J.C. & Bertness, M.D. (2006) Mechanisms of exclusion of native coastal marsh plants by an invasive grass. *Journal of Ecology,* 94, 342-354.

Minden, V., Jacobi, J.D., Porembski, S. & Boehmer, H.J. (2010) Effects of invasive alien kahili ginger (*Hedychium gardnerianum*) on native plant species regeneration in a Hawaiian rainforest. *Applied Vegetation Science*, 13, 5-14.

Monty, A., Stainier, C., Lebeau, F., Pieret, N. & Mahy, G. (2008) Seed rain pattern of the invasive weed *Senecio inaequidens* (Asteraceae). *Belgian Journal of Botany,* 141, 51-63.

Morgan, E.C. & Overholt, W.A. (2005) Potential allelopathic effects of Brazilian pepper (*Schinus terebinthifolius* Raddi, Anacardiaceae) aqueous extract on germination and growth of selected Florida native plants. *Journal of the Torrey Botanical Society,* 132, 11-15.

Muchovej, J.J., Onokpise, O.U. & Bambo, S.K. (2009) Characteristics of cogon grass rhizomes and its perforation of a maiden cane rhizome. *International Journal of Botany,* 5, 314-316.

Mummey, D.L. & Rillig, M.C. (2006). The invasive plant species *Centaurea maculosa* alters arbuscular mycorrhizal fungal communities in the field. *Plant and Soil,* 288, 81-90.

Mummey, D.L., Rillig, M.C. & Holben, W.E. (2005) Neighboring plant influences on arbuscular mycorrhizal fungal community composition as assessed by T-RFLP analysis. *Plant and Soil,* 271, 83-90.

Munoz, A.A. & Cavieres, L.A. (2008) The presence of a showy invasive plant disrupts pollinator service and reproductive output in native alpine species only at high densities. *Journal of Ecology,* 96, 459-467.

Murrell, C., Gerber, E., Krebs, C., Parepa, M., Schaffner, U. & Bossdorf, O. (2011) Invasive knotweed affects native plants through allelopathy. *American Journal of Botany,* 98, 38-43.

Musil, C.F. (1993) Effect of invasive Australian acacias on the regeneration, growth, and nutrient chemistry of South African lowland fynbos. *Journal of Applied Ecology,* 30, 361-372.

Musil, C.F. & Midgley, G.F. (1990) The relative impact of invasvie Australian acacias, fire and season on the soil chemical status of a sand plain lowland fynbos community. *South African Journal of Botany,* 56, 419-427.

Nilsson, C., Engelmark, O., Cory, J., Forsslund, A. & Carlborg, E. (2008) Differences in litter cover and understorey flora between stands of introduced lodgepole pine and native scots pine in Sweden. *Forest Ecology and Management,* 255, 1900-1905.

Niu, H.-b., Liu, W.-x., Wan, F.-h. & Liu, B. (2007) An invasive aster (*Ageratina adenophora*) invades and dominates forest understories in China: altered soil microbial communities facilitate the invader and inhibit natives. *Plant and Soil,* 294, 73-85.

Ogden, J.A.E. & Rejmánek, M. (2005). Recovery of native plant communities after the control of a dominant invasive plant species, *Foeniculum vulgare*: Implications for management. *Biological Conservation,* 125, 427-439.

Ogle, S.M., Reiners, W.A. & Gerow, K.G. (2003) Impacts of exotic annual brome grasses (*Bromus* spp.) on ecosystem properties of northern mixed grass prairie. *American Midland Naturalist,* 149, 46-58.

Orr, S.P., Rudgers, J.A. & Clay, K. (2005) Invasive plants can inhibit native tree seedlings: testing potential allelopathic mechanisms. *Plant Ecology,* 181, 153-165.

Orrock, J.L., Witter, M.S. & Reichman, O.J. (2008) Apparent competition with an exotic plant reduces native plant establishment. *Ecology,* 89, 1168-1174.

Ortega, Y.K., McKelvey, K.S. & Six, D.L. (2006) Invasion of an exotic forb impacts reproductive success and site fidelity of a migratory songbird. *Oecologia,* 149, 340-351.

Ortega, Y.K. & Pearson, D.E. (2005) Weak vs. strong invaders of natural plant communities: Assessing invasibility and impact. *Ecological Applications,* 15, 651-661.

Osland, M.J., González, E., Richardson, C.J. (2011) Restoring diversity after cattail expansion: disturbance, resilience, and seasonality in a tropical dry wetland. *Ecological Applications,* 21, 715-728.

Osland, M.J., Pahl, J.W. & Richardson, C.J. (2009) Native bamboo [*Arundinaria gigantea* (Walter) Muhl., Poaceae] establishment and growth after the removal of an invasive non-native shrub (*Ligustrum sinense* Lour., Oleaceae): Implications for Restoration. *Castanea,* 74, 247-258.

Oswalt, C.M., Oswalt, S.N. & Clatterbuck, W.K. (2007) Effects of *Microstegium vimineum* (Trin.) A. Camus on native woody species density and diversity in a productive mixed-hardwood forest in Tennessee. *Forest Ecology and Management,* 242, 727-732.

Otto, S., Groffman, P.M., Findlay, S.E.G. & Arreola, A.E. (1999) Invasive plant species and microbial processes in a tidal freshwater marsh. *Journal of Environmental Quality,* 28, 1252-1257.

Padrón, B., Nogales, M., Traveset, A, Vilà, M., Martínez-Abraín, A., Padilla, D.P. & Marrero, P. (2011). Integration of invasive *Opuntia* spp. by native and alien seed dispersers in the Mediterranean area and the Canary Islands. *Biological Invasions,* 13, 831-844.

Paterson, I.D., Coetzee, J.A. Hill, M.P. & Downie, D.D. (2011) A pre-release assessment of the relationship between the invasive alien plant, *Pereskia aculeata* Miller (Cactaceae), and native plant biodiversity in South Africa. *Biological Control,* 57, 59-65.

Patten, K. & O'Casey, C. (2007). Use of Willapa Bay, Washington, by shorebirds and waterfowl after *Spartina* control efforts. *Journal of Field Ornithology,* 78, 395-400.

Pauchard, A., García, R.A., Peña, E., González, C., Lohengrin, L.A., & Bustamante, R.O. (2008) Positive feedbacks between plant invasions and fire regimes: *Teline monspessulana* (L.) K. Koch (Fabaceae) in central Chile. *Biological Invasions*, 10, 547-553.

Pearson, D.E. (2009) Invasive plant architecture alters trophic interactions by changing predator abundance and behavior. *Oecologia,* 159, 549-558.

Peng, R.H., Fang, C.M., Li, B. & Chen, J.K. (2011). *Spartina alterniflora* invasion increases soil inorganic nitrogen pools through interactions with tidal subsidies in the Yangtze Estuary, China. *Oecologia,* 165, 797-807.

Pétillon, J., Lambeets, K., Montaigne, W., Maelfait, J.P. & Bonte, D. (2010) Habitat structure modified by an invasive grass enhances inundation withstanding in a salt-marsh wolf spider. *Biological Invasions,* 12, 3219-3226.

Pétillon, J., Lasne, E., Lambeets, K., Canard, A., Vernon, P. & Ysnel, F. (2010) How do alterations in habitat structure by an invasive grass affect salt-marsh resident spiders? *Annales Zoologici Fennici,* 47, 79-89.

Pétillon, J., Puzin, C., Acou, A. & Outreman, Y. (2009) Plant invasion phenomenon enhances reproduction performance in an endangered spider. *Naturwissenschaften,* 96, 1241-1246.

Pétillon, J., Ysnel, F., Canard, A. & Lefeuvre, J.C. (2005) Impact of an invasive plant (*Elymus athericus*) on the conservation value of tidal salt marshes in western France and implications for management: Responses of spider populations. *Biological Conservation,* 126, 103-117.

Petsikos, C., Dalias, P. & Troumbis, A.Y. (2007) Effects of *Oxalis pes-caprae* L. invasion in olive groves. *Agriculture Ecosystems & Environment,* 120, 325-329.

Pickart, A.J., Theiss, K.C., Stauffer, H.B. & Olsen, G.T. (1998) Yellow bush lupine invasion in northern California coastal dunes - II. Mechanical restoration techniques. *Restoration Ecology,* 6, 69-74.

Potts, D.L., Harpole, W.S., Goulden, M.L. & Suding, K.N. (2008) The impact of invasion and subsequent removal of an exotic thistle, *Cynara cardunculus*, on CO_2_ and H_2_O vapor exchange in a coastal California grassland. *Biological Invasions,* 10, 1073-1084.

Prasad, A.E. (2010) Effects of an exotic plant invasion on native understory plants in a tropical dry forest. *Conservation Biology,* 24, 747-757.

Pritekel, C., Whittemore-Olson, A., Snow, N. & Moore, J.C. (2006) Impacts from invasive plant species and their control on the plant community and belowground ecosystem at Rocky Mountain National Park, USA. *Applied Soil Ecology,* 32, 132-141.

Provencher, L., Herring, B.J., Gordon, D.R., Rodgers, H.L., Tanner, G.W., Brennan, L.A. & Hardesty, J.L. (2000) Restoration of northwest Florida sandhills through harvest of invasive *Pinus clausa*. *Restoration Ecology,* 8, 175-185.

Rahlao, S.J., Milton, S.J., Esler, K.J., Van Wilgen, B.W. & Barnard, P. (2009) Effects of invasion of fire-free arid shrublands by a fire-promoting invasive alien grass (*Pennisetum setaceum*) in South Africa. *Austral Ecology,* 34, 920-928.

Ramos, J.A. (1996) Introduction of exotic tree species as a threat to the Azores bullfinch population. *Journal of Applied Ecology,* 33, 710-722.

Rand, T.A. & Louda S.M. (2004) Exotic weed invasion increases the susceptibility of native plants attack by a biocontrol herbivore. *Ecology,* 85, 1548-1554.

Reed, H.E., Seastedt, T.R. & Blair, J.M. (2005) Ecological consequences of C-4 grass invasion of a C-4 grassland: A dilemma for management. *Ecological Applications,* 15, 1560-1569.

Reinhart, K.O., Greene, E. & Callaway, R.M. (2005) Effects of *Acer platanoides* invasion on understory plant communities and tree regeneration in the northern Rocky Mountains. *Ecography,* 28, 573-582.

Reinhart, K.O. & Rinella, M. (2011) Comparing susceptibility of eastern and western US grasslands to competition and allelopathy from spotted knapweed *Centaurea stoebe* L. subsp *micranthos* (Gugler) Hayek. *Plant Ecology* 212, 821-828.

Renne, I.J., Rios, B.G., Fehmi, J.S. & Tracy, B.F. (2004) Low allelopathic potential of an invasive forage grass on native grassland plants: a cause for encouragement? *Basic and Applied Ecology,* 5, 261-269.

Rhoades, C., Barnes, T. & Washburn, B. (2002) Prescribed fire and herbicide effects on soil processes during barrens restoration. *Restoration Ecology,* 10, 656-664.

Rice, S.K., Westerman, B. & Federici, R. (2004) Impacts of the exotic, nitrogen-fixing black locust (*Robinia pseudoacacia*) on nitrogen-cycling in a pine-oak ecosystem. *Plant Ecology,* 174, 97-107.

Richburg, J.A., Patterson, W.A. & Lowenstein, F. (2001) Effects of road salt and *Phragmites australis* invasion on the vegetation of a western Massachusetts calcareous lake-basin fen. *Wetlands,* 21, 247-255.

Rodewald, A.D., Shustack, D.P. & Hitchcok, L.E. (2010) Exotic shrubs as ephemeral ecological traps for nesting birds. *Biological Invasions,* 12, 33-39.

Rodgers, V.L., Wolfe, B.E., Werden, L.K. & Finzi, A.C. (2008) The invasive species *Alliaria petiolata* (garlic mustard) increases soil nutrient availability in northern hardwood-conifer forests. *Oecologia,* 157, 459-471.

Rosas, H.L., Moreno-Casasola, P. & Mendelssohn, I.A. (2006) Effects of experimental disturbances on a tropical freshwater marsh invaded by the African grass *Echinochloa pyramidalis*. *Wetlands,* 26, 593-604.

Rose, S. & Fairweather, P. G. (1997) Changes in floristic composition of urban bushland invaded by *Pittosporum undulatum* in northern Sydney, Australia. *Australian Journal of Botany,* 45, 123-149.

Rossiter, N., Setterfield, S., Douglas, M., Hutley, L. & Cook, G. (2004) Exotic grass invasion in the tropical savanna of northern Australia: ecosystem consequences. *Proceedings of the 14th Australian weeds conference* (ed. by B.M. Sindel & S. B. Johnson), pp. 168–71. Weed Society of New South Wales, Sydney.

Rothstein, D.E., Vitousek, P.M. & Simmons, B.L. (2004) An exotic tree alters decomposition and nutrient cycling in a Hawaiian montane forest. *Ecosystems,* 7, 805-814.

Rudgers, J.A. & Orr, S. (2009) Non-native grass alters growth of native tree species via leaf and soil microbes. *Journal of Ecology,* 97, 247-255.

Saggar, S., McIntosh, P.D., Hedley, C.B. & Knicker, H. (1999) Changes in soil microbial biomass, metabolic quotient, and organic matter turnover under *Hieracium* (*H. pilosella* L.). *Biology and Fertility of Soils,* 30, 232-238.

Sanon, A., Béguiristain, T., Cébron, A., Berthelin, J., Ndoye, I., Leyval, C., Sylla, S. & Duponnois, R. (2009) Changes in soil diversity and global activities following invasions of the exotic invasive plant, *Amaranthus viridis* L., decrease the growth of native sahelian *Acacia* species. *Fems Microbiology Ecology,* 70, 118-131.

Scheiman, D.M., Bollinger, E.K. & Johnson, D.H. (2003) Effects of leafy spurge infestation on grassland birds. *Journal of Wildlife Management,* 67, 115-121.

Schirmel, J., Timler, L. & Buchholz, S. (2011) Impact of the invasive moss *Campylopus introflexus* on carabid beetles (Coleoptera: Carabidae) and spiders (Araneae) in acidic coastal dunes at the southern Baltic Sea. *Biological Invasions,* 13, 605-620.

Schlossberg, S. & King, D.I. (2010) Effects of invasive woody plants on avian nest site selection and nesting success in shrublands. *Animal Conservation,* 13, 286-293.

Schmidt, K.A., Nelis, L.C., Briggs, N. & Ostfeld, R.S. (2005). Invasive shrubs and songbird nesting success: Effects of climate variability and predator abundance. *Ecological Applications,* 15, 258-265.

Schmidt, K.A. & Whelan C.J. (1999) Effects of exotic *Lonicera* and *Rhamnus* on songbird nest predation. *Conservation Biology,* 13, 1502-1506.

Schooler, S.S., Cook, T., Prichard, G. & Yeates, A.G. (2010) Disturbance-mediated competition: the interacting roles of inundation regime and mechanical and herbicidal control in determining native and invasive plant abundance. *Biological Invasions,* 12, 3289-3298.

Schooler, S.S., McEvoy, P.B. & Coombs, E.M. (2006) Negative per capita effects of purple loosestrife and reed canary grass on plant diversity of wetland communities. *Diversity and Distributions,* 12, 351-363.

Schooler, S.S., McEvoy, P.B., Hammond, P. & Coombs, E.M. (2009) Negative per capita effects of two invasive plants, *Lythrum salicaria* and *Phalaris arundinacea*, on the moth diversity of wetland communities. *Bulletin of Entomological Research,* 99, 229-243.

Schooler, S.S., Yeates, A.G., Wilson, J.R.U. & Julien, M.H. (2007) Herbivory, mowing, and herbicides differently affect production and nutrient allocation of *Alternanthera philoxeroides*. *Aquatic Botany,* 86, 62-68.

Schutzenhofer, M.R. & Valone T.J. (2006) Positive and negative effects of exotic *Erodium cicutarium* on an arid ecosystem. *Biological Conservation,* 132, 376-381.

Shaben, J. & Myers J.H. (2010) Relationships between Scotch broom (*Cytisus scoparius*), soil nutrients, and plant diversity in the Garry oak savannah ecosystem. *Plant Ecology,* 207, 81-91.

Sher, A.A. & Marshall, D.L. (2003) Seedling competition between native *Populus deltoides* (Salicaceae) and exotic *Tamarix ramosissima* (Tamaricaceae) across water regimes and substrate types. *American Journal of Botany,* 90, 413-422.

Siemens, T.J. & Blossey, B. (2007) An evaluation of mechanisms preventing growth and survival of two native species in invasive bohemian knotweed (*Fallopia* x *bohemica*, Polygonaceae). *American Journal of Botany,* 94, 776-783.

Simao, M.C.M., Flory, S.L. & Rudgers, J.A. (2010) Experimental plant invasion reduces arthropod abundance and richness across multiple trophic levels. *Oikos,* 119, 1553-1562.

Slobodchikoff, C.N. & Doyen, J.T. (1977) Effects of *Ammophila arenaria* on sand dune arthropod communities. *Ecology,* 58, 1171-1175.

Small, C.J., White, D.C. & Hargbol, B. (2010) Allelopathic influences of the invasive *Ailanthus altissima* on a native and a non-native herb. *Journal of the Torrey Botanical Society,* 137, 366-372.

Smoliak, S. & Dormaar, J.F. (1985) Productivity of Russian wildrye and crested wheat grass and their effect on prairie soils. *Journal of Range Management,* 38, 403-405.

Spellman, B.T. & Wurtz, T.L. (2011) Invasive sweetclover (*Melilotus alba*) impacts native seedling recruitment along floodplains of interior Alaska. *Biological Invasions,* 13, 1779-1790.

Sperry, L.J., Belnap, J. & Evans, R.D. (2006) *Bromus tectorum* invasion alters nitrogen dynamics in an undisturbed arid grassland ecosystem. *Ecology,* 87, 603-615.

St. John, M.G., Wall, D.H. & Hunt, H.W. (2006) Are soil mite assemblages structured by the identity of native and invasive alien grasses? *Ecology,* 87, 1314-1324.

Standish, R.J. (2004) Impact of an invasive clonal herb on epigaeic invertebrates in forest remnants in New Zealand. *Biological Conservation,* 116, 49-58.

Standish, R.J., Robertson, A.W. & Williams, P.A. (2001) The impact of an invasive weed *Tradescantia fluminensis* on native forest regeneration. *Journal of Applied Ecology,* 38, 1253-1263.

Standish, R.J., Williams, P.A., Robertson, A.W., Scott, N.A. & Hedderley, D.I. (2004) Invasion by a perennial herb increases decomposition rate and alters nutrient availability in warm temperate lowland forest remnants. *Biological Invasions,* 6, 71-81.

Stanley, A.G., Dunwiddie, P.W. & Kaye, T.N. (2011) Restoring invaded Pacific Northwest prairies: Management recommendations from a region-wide experiment. *Northwest Science,* 85, 233-246.

Steenkamp, H.E. & Chown, S.L. (1996) Influence of dense stands of an exotic tree, *Prosopis glandulosa* Benson, on a savanna dung beetle (Coleoptera: Scarabaeinae) assemblage in southern Africa. *Biological Conservation,* 78, 305-311.

Steers, R.J. & Allen, E.B. (2010) Post-fire control of invasive plants promotes native recovery in a burned desert shrubland. *Restoration Ecology,* 18, 334-343.

Stinson, K.A., Campbell, S.A., Powell, J.R., Wolfe, B.J., Callaway, R. M., Thelen, G.C., Hallett, S.G., Prati, D., & Klironomos, J.N. (2006) Invasive plant suppresses growth of native tree seedlings by disrupting belowground mutualisms. *PLOS Biology*, 4, 727-731.

Stock, W.D., Wienand, K.T. & Baker, A.C. (1995) Impacts of invading N_2_-fixing *Acacia* species on patterns of nutrient cycling in two cape ecosystems: evidence from soil incubation studies and ^15^N natural abundance values. *Oecologia,* 101, 375-382.

Strickland, M.S., Devore, J.L., Maerz, J.C. & Bradford, M.A. (2010) Grass invasion of a hardwood forest is associated with declines in belowground carbon pools. *Global Change Biology,* 16, 1338-1350.

Strickland, M.S., DeVore, J.L., Maerz, J.C. & Bradford, M.A. (2011) Loss of faster-cycling soil carbon pools following grass invasion across multiple forest sites. *Soil Biology & Biochemistry,* 43, 452-454.

Suding, K.N., LeJeune, K.D. & Seastedt, T.R. (2004) Competitive impacts and responses of an invasive weed: dependencies on nitrogen and phosphorus availability. *Oecologia,* 141, 526-535.

Svejcar, T. & Sheley, R. (2001) Nitrogen dynamics in perennial- and annual-dominated arid rangeland. *Journal of Arid Environments,* 47, 33-46.

Swab, R.M., Zhang, L. & Mitsch, W.J. (2008) Effect of hydrologic restoration and *Lonicera maackii* removal on herbaceous understory vegetation in a bottomland hardwood forest. *Restoration Ecology,* 16, 453-463.

Takahashi, M., Giambelluca, T.W., Mudd, R.G., DeLay, J.K., Nullett, M.A. & Asner, G.P. (2011) Rainfall partitioning and cloud water interception in native forest and invaded forest in Hawai'i Volcanoes National Park. *Hydrological Processes,* 25, 448-464.

Thomas, C.D., Ng, D., Singer, M.C., Mallet, J.L.B, Parmesan, C. & Billington, H.L. (1987) Incorporation of a European weed into the diet of a North American herbivore. *Evolution,* 41, 892-901.

Thorpe, A.S., Archer, V. & DeLuca, T.H. (2006) The invasive forb, *Centaurea maculosa*, increases phosphorus availability in Montana grasslands. *Applied Soil Ecology,* 32, 118-122.

Toft, R.J., Harris, R.J. and Williams, P.A. (2001) Impacts of the weed *Tradescantia fluminensis* on insect communities in fragmented forests in New Zealand. *Biological Conservation,* 102, 31-46.

Trammell, M.A. & Butler, J.L. (1995) Effects of exotic plants on native ungulate use of habitat. *Journal of Wildlife Management,* 59, 808-816.

Treberg, M.A. & Husband, B.C. (1999) Relationship between the abundance of *Lythrum salicaria* (purple loosestrife) and plant species richness along the Bar River, Canada. *Wetlands,* 19, 118-125.

Truscott, A.M., Palmer, S.C., Soulsby, C., Westaway, S. & Hulme, P.E. (2008) Consequences of invasion by the alien plant *Mimulus guttatus* on the species composition and soil properties of riparian plant communities in Scotland. *Perspectives in Plant Ecology Evolution and Systematics,* 10, 231-240.

Tsai, J.K., Sun, H.T., Chen, C.F., Hsieh, C.F. & Wu, S.H. (2010) The impact of naturalized legumes on plant communities in Northern Taiwan: are we worrying too much? *Plant Ecology,* 211, 171-180.

Tscheulin, T., Petanidou, T., Potts, S.G. & Settele, J. (2009) The impact of *Solanum elaeagnifolium*, an invasive plant in the Mediterranean, on the flower visitation and seed set of the native co-flowering species *Glaucium flavum*. *Plant Ecology,* 205, 77-85.

Turnbull, LA., Rahm, S., Baudois, O., Eichenberger-Glinz, S., Wacker, L. & Schmid, B. (2005) Experimental invasion by legumes reveals non-random assembly rules in grassland communities. *Journal of Ecology,* 93, 1062-1070.

Turner, P.J., Scott, J.K. & Spafford, H. (2008) The ecological barriers to the recovery of bridal creeper (*Asparagus asparagoides* (L.) Druce) infested sites: Impacts on vegetation and the potential increase in other exotic species. *Austral Ecology,* 33, 713-722.

Turner, P.J., Scott, J.K. & Spafford, H. (2011) Bridal creeper (*Asparagus asparagoides*)-invaded sites with elevated levels of available soil nutrients: barrier to restoration? *Invasive Plant Science and Management,* 4, 212-222.

Tuttle, N.C., Beard, K.H. & Pitt, W.C. (2009) Invasive litter, not an invasive insectivore, determines invertebrate communities in Hawaiian forests. *Biological Invasions,* 11, 845-855.

Tyrer, S.J., Hild, A.L., Mealor, B.A. & Munn, L.C. (2007) Establishment of native species in soils from Russian knapweed (*Acroptilon repens*) invasions. *Rangeland Ecology & Management,* 60, 604-612.

Valery, L., Bouchard, V., & Lefeuvre, J.C. (2004) Impact of the invasive native species *Elymus athericus* on carbon pools in a salt marsh. *Wetlands,* 24, 268-276.

Valtonen, A., Jantunen, J. & Saarinen, K. (2006) Flora and lepidoptera fauna adversely affected by invasive *Lupinus polyphyllus* along road verges. *Biological Conservation,* 133, 389-396.

van der Wal, R., Truscott, A.M. , Pearce, I.S.K., Cole, L., Harris, M.P. & Wanless, S. (2008) Multiple anthropogenic changes cause biodiversity loss through plant invasion. *Global Change Biology,* 14, 1428-1436.

Van Riper, L.C. & Larson D.L. (2009) Role of invasive *Melilotus officinalis* in two native plant communities. *Plant Ecology,* 200, 129-139.

Vanderhoeven, S., Dassonville, N., Chapuis-Lardy, L., Hayez, M. & Meerts, P. (2006) Impact of the invasive alien plant *Solidago gigantea* on primary productivity, plant nutrient content and soil mineral nutrient concentrations. *Plant and Soil,* 286, 259-268.

Vanderhoeven, S., Dassonville, N. & Meerts, P. (2005) Increased topsoil mineral nutrient concentrations under exotic invasive plants in Belgium. *Plant and Soil,* 275, 169-179.

Vanwilgen, B.W. & Richardson D.M. (1985) The effects of alien shrub invasions on vegetation structure and fire behavior in South African fynbos shrublands - a simulation study. *Journal of Applied Ecology,* 22, 955-966.

Vilà, M., Tessier, M., Suehs, C.M., Brundu, G., Carta, L., Galanidis, A., Lambdon, P., Manca, M., Médail, F., Moragues, E. Traveset, A., Troumbis, A.Y. & Hulme, P.E. (2006) Local and regional assessments of the impacts of plant invaders on vegetation structure and soil properties of Mediterranean islands. *Journal of Biogeography,* 33, 853-861.

Vitousek, P.M. & Walker, L.R. (1989) Biological invasion by *Myrica faya* in Hawaii - plant demography, nitrogen fixation, ecosystem effects. *Ecological Monographs,* 59, 247-265.

Vitousek, P.M., Walker, L.R., Whiteaker, L.D., Mueller-Dombois, D. & Matson, P.A. (1987) Biological invasion by *Myrica faya* alters ecosystem development in Hawaii. *Science,* 238, 802-804.

Vivrette, N.J. & Muller C.H. (1977) Mechanism of invasion and dominance of coastal grassland by *Mesembryanthemum crystallinum*. *Ecological Monographs,* 47, 301-318.

Von Holle, B., Joseph, K.A., Largay, E.F. & Lohnes, R.G. (2006) Facilitations between the introduced nitrogen-fixing tree, *Robinia pseudoacacia*, and nonnative plant species in the glacial outwash upland ecosystem of cape cod, MA. *Biodiversity and Conservation,* 15, 2197-2215.

Walker, L.R. & Vitousek, P.M. (1991). An invader alters germination and growth of a native dominant tree in Hawaii. *Ecology,* 72, 1449-1455.

Wan-Xue, L., Hong-Bang, N., Fang-Haoo, W., & Bo, L. (2010) Effects of leachates of the invasive plant, *Ageratina adenophora* (Sprengel) on soil microbial community. *Acta Ecologica Sinica*, 30, 196-200.

Wardle, D.A., Nicholson, K.S., Ahmed, M. & Rahman, A. (1994) Interference effects of the invasive plant *Carduus nutans* L against the nitrogen fixation ability of *Trifolium repens* L. *Plant and Soil,* 163, 287-297.

Wardle, D.A., Nicholson, K.S.& Rahman, A. (1995) Ecological effects of the invasive weed species *Senecio jacobaea* L (ragwort) in a New Zealand pasture. *Agriculture Ecosystems & Environment,* 56, 19-28.

Watling, J.I., Hickman, C.R., Lee, E., Wang, K. & Orrock, J.L. (2011) Extracts of the invasive shrub *Lonicera maackii* increase mortality and alter behavior of amphibian larvae. *Oecologia,* 165, 153-159.

Watling, J.I., Hickman, C.R. & Orrock, J.L. (2011) Invasive shrub alters native forest amphibian communities. *Biological Conservation,* 144, 2597-2601.

Wearne, L.J. & Morgan J.W. (2004) Community-level changes in Australian subalpine vegetation following invasion by the non-native shrub *Cytisus scoparius*. *Journal of Vegetation Science,* 15, 595-604.

Webb, S.L., Pendergast, T.H. & Dwyer, M.E. (2001) Response of native and exotic maple seedling banks to removal of the exotic, invasive Norway maple (*Acer platanoides*). *Journal of the Torrey Botanical Society,* 128, 141-149.

Wedin, D.A. & Pastor, J. (1993) Nitrogen mineralization dynamics in grass monocultures. *Oecologia,* 96, 186-192.

Wilcox, J. & Beck, C.W. (2007) Effects of *Ligustrum sinense* Lour. (Chinese privet) on abundance and diversity of songbirds and native plants in a southeastern nature preserve. *Southeastern Naturalist,* 6, 535-550.

Wilkie, L., Cassis, G. & Gray, M. (2007) The effects on terrestrial arthropod communities of invasion of a coastal heath ecosystem by the exotic weed bitou bush (*Chrysanthemoides monilifera* ssp *rotundata* L.). *Biological Invasions,* 9, 477-498.

Williams, J.L. & Crone, E.E. (2006) The impact of invasive grasses on the population growth of *Anemone patens*, a long-lived native forb. *Ecology,* 87, 3200-3208.

Williams, K., Westrick, L.J. & Williams, B.J. (2006) Effects of blackberry (*Rubus discolor*) invasion on oak population dynamics in a California savanna. *Forest Ecology and Management,* 228, 187-196.

Williams, M.C. & Wardle, G.M. (2007) Pine and eucalypt litterfall in a pine-invaded eucalypt woodland: The role of fire and canopy cover. *Forest Ecology and Management,* 253, 1-10.

Wilson, J.R.U., Yeates, A., Schooler, S. & Julien, M.H. (2007) Rapid response to shoot removal by the invasive wetland plant, alligator weed (*Alternanthera philoxeroides*). *Environmental and Experimental Botany,* 60, 20-25.

Windham, L. (2001) Comparison of biomass production and decomposition between *Phragmites australis* (common reed) and *Spartina patens* (salt hay grass) in brackish tidal marshes of New Jersey, USA. *Wetlands,* 21, 179-188.

Windham, L. & Ehrenfeld, J.G. (2003). Net impact of a plant invasion on nitrogen-cycling processes within a brackish tidal marsh. *Ecological Applications,* 13, 883-896.

Windham, L. & Lathrop, R.G. (1999) Effects of *Phragmites australis* (common reed) invasion on aboveground biomass and soil properties in brackish tidal marsh of the Mullica River, New Jersey. *Estuaries,* 22, 927-935.

Witkowski, E.T.F. (1991) Growth and competition between seedlings of *Protea repens* (L) and the alien invasive, *Acacia saligna* (Labill) Wendl in relation to nutrient availability. *Functional Ecology,* 5, 101-110.

Witkowski, E.T.F. (1991) Effects of invasive alien *Acacias* on nutrient cycling in the coastal lowlands of the Cape fynbos. *Journal of Applied Ecology,* 28, 1-15.

Wixted, K.L. & McGraw, J.B. (2010). Competitive and allelopathic effects of garlic mustard (*Alliaria petiolata*) on American ginseng (*Panax quinquefolius*). *Plant Ecology,* 208, 347-357.

Wolf, J.J., Beatty, S.W. & Seastedt, T.R. (2004) Soil characteristics of Rocky Mountain National Park grasslands invaded by *Melilotus officinalis* and *M. alba*. *Journal of Biogeography,* 31, 415-424.

Wolkovich, E.M., Bolger, D.T. & Cottingham, K.L. (2009) Invasive grass litter facilitates native shrubs through abiotic effects. *Journal of Vegetation Science,* 20, 1121-1132.

Woods, K.D. (1993) Effects of invasion by *Lonicera tatarica* L on herbs and tree seedlings in four New England forests. *American Midland Naturalist,* 130, 62-74.

Wootton, L.S., Halsey, S.D., Bevaart, K., McGough, A., Ondreicka, J. & Patel, P. (2005) When invasive species have benefits as well as costs: managing *Carex kobomugi* (Asiatic sand sedge) in New Jersey's coastal dunes. *Biological Invasions,* 7, 1017-1027.

Wyckoff, P.H. & Webb, S.L. (1996) Understory influence of the invasive Norway maple (*Acer platanoides*). *Bulletin of the Torrey Botanical Club,* 123, 197-205.

Yang, R.Y., Mei, L.X., Tang, J.J. & Chen, X. (2007) Allelopathic effects of invasive *Solidago canadensis* L. on germination and growth of native Chinese plant species. *Allelopathy Journal,* 19, 241-247.

Yeates, A.G. & Schooler, S.S. (2011). Influence of *Lantana camara* and its removal on tree dynamics in a recently burnt wet Sclerophyll forest in Northern NSW. *Ecological Management & Restoration,* 12, 236-241.

Yelenik, S.G., Stock, W.D. & Richardson, D.M. (2007) Functional group identity does not predict invader impacts: differential effects of nitrogen-fixing exotic plants on ecosystem function. *Biological Invasions,* 9, 117-125.

Yoshida, K. & Oka, S. (2000) Impact of biological invasion of *Leucaena leucocephala* on successional pathway and species diversity of secondary forest on Hahajima Island, Ogasawara (Bonin) Islands, northwestern Pacific. *Japanese Journal of Ecology,* 50, 111-119.

Young, J.A. & Evans, R.A. (1971) Medusahead invasion as influenced by herbicides and grazing on low sagebrush sites. *Journal of Range Management,* 24, 451-&.

Zhang, C.B., Wang, J., Qian, B.Y. & Li, W.H. (2009) Effects of the invader *Solidago canadensis* on soil properties. *Applied Soil Ecology,* 43, 163-169.

Zhang, S., Jin, Y., Tang, J. & Chen, X. (2009) The invasive plant *Solidago canadensis* L. suppresses local soil pathogens through allelopathy. *Applied Soil Ecology,* 41, 215-222.

Zhi, Y., Li, H., An, S., Zhao, L., Zhou, C. & Deng, Z. (2007) Inter-specific competition: *Spartina alterniflora* is replacing *Spartina anglica* in coastal China. *Estuarine Coastal and Shelf Science,* 74, 437-448.
